# Diffuse optical reconstructions of functional near infrared spectroscopy data using maximum entropy on the mean

**DOI:** 10.1038/s41598-022-06082-1

**Published:** 2022-02-10

**Authors:** Zhengchen Cai, Alexis Machado, Rasheda Arman Chowdhury, Amanda Spilkin, Thomas Vincent, Ümit Aydin, Giovanni Pellegrino, Jean-Marc Lina, Christophe Grova

**Affiliations:** 1 Department of Physics and PERFORM Centre, Concordia University, Montreal, Canada; 2 Multimodal Functional Imaging Lab, Biomedical Engineering Department, McGill University, Montreal, Canada; 3 Neurology and Neurosurgery Department, Montreal Neurological Institute, McGill University, Montreal, Canada; 4 Centre de médecine préventive et d’activité physique, Montréal Heart Institute, Montréal, Canada; 5 MRC Social, Genetic and Developmental Psychiatry Centre, Institute of Psychiatry, Psychology and Neuroscience, King’s College London, London, UK; 6 École de technologie supérieure de l’Université du Québec, Montréal, Canada; 7 Centre de Recherches Mathématiques, Université de Montréal, Montréal, Canada

**Keywords:** Biomedical engineering, Biophysical models

## Abstract

Functional near-infrared spectroscopy (fNIRS) measures the hemoglobin concentration changes associated with neuronal activity. Diffuse optical tomography (DOT) consists of reconstructing the optical density changes measured from scalp channels to the oxy-/deoxy-hemoglobin concentration changes within the cortical regions. In the present study, we adapted a nonlinear source localization method developed and validated in the context of Electro- and Magneto-Encephalography (EEG/MEG): the Maximum Entropy on the Mean (MEM), to solve the inverse problem of DOT reconstruction. We first introduced depth weighting strategy within the MEM framework for DOT reconstruction to avoid biasing the reconstruction results of DOT towards superficial regions. We also proposed a new initialization of the MEM model improving the temporal accuracy of the original MEM framework. To evaluate MEM performance and compare with widely used depth weighted Minimum Norm Estimate (MNE) inverse solution, we applied a realistic simulation scheme which contained 4000 simulations generated by 250 different seeds at different locations and 4 spatial extents ranging from 3 to 40$\text {cm}^2$ along the cortical surface. Our results showed that overall MEM provided more accurate DOT reconstructions than MNE. Moreover, we found that MEM was remained particularly robust in low signal-to-noise ratio (SNR) conditions. The proposed method was further illustrated by comparing to functional Magnetic Resonance Imaging (fMRI) activation maps, on real data involving finger tapping tasks with two different montages. The results showed that MEM provided more accurate HbO and HbR reconstructions in spatial agreement with the main fMRI cluster, when compared to MNE.

## Introduction

Functional Near-infrared spectroscopy (fNIRS) is an non-invasive functional neuroimaging modality. It detects changes in oxy-/deoxy-hemoglobin (i.e., HbO/HbR) concentration within head tissues through the measurement of near-infrared light absorption using sources and detectors placed on the surface of the head^1,2^. In continuous wave fNIRS, the conventional way to transform variations in optical density to HbO/HbR concentration changes at the level of each source-detector channel, is to apply the modified Beer Lambert Law (mBLL)^3^. This model assumes homogeneous concentration changes within the detecting region, i.e., ignoring the partial volume effects which indicates the absorption of light within the illuminated regions varies locally. This assumption reduces quantitative accuracy of HbO/HbR concentration changes when dealing with focal hemodynamic changes^4,5^.

In order to handle these important quantification biases associated with sensor level based analysis, diffuse optical tomography (DOT) has been proposed to reconstruct, from sensor level measures of the optical density, the fluctuations of HbO/HbR concentrations within the brain^6^. This technique not only provides better spatial localization accuracy and resolution of the underlying hemodynamic responses^7,8^, but also avoids partial volume effect in classical mBLL, hence achieves better quantitative estimation of HbO/HbR concentration changes^4,5^. DOT has been applied to reconstruct hemodynamic responses in sensory and motor cortex during median-nerve stimulation^9,10^ and finger tapping^7,11^; to conduct visual cortex retinotopic mapping^12–14^ and to simultaneous image hemodynamic responses over the motor and visual cortex^15^.

To formalize DOT reconstruction, one needs to solve two main problems. The first one is the forward problem which estimates a forward model or sensitivity matrix that maps local absorption changes within the brain to variations of optical density changes measured by each channel^16^. The second problem is the inverse problem which aims at reconstructing the fluctuations of hemodynamic activity within the brain from scalp measurements^17^. The forward problem can be solved by generating a subject specific anatomical model, describing accurately propagation of light within the head. Such anatomical model is obtained by segmenting anatomical Magnetic Resonance Imaging (MRI) data, typically into five tissues (i.e., scalp, skull, cerebro-spinal fluid (CSF), white matter and gray matter), before initializing absorption and scattering coefficients values for each tissue type and for each wavelength^18,19^. Solving the inverse problem relies on solving an ill-posed problem which does not provide a unique solution, unless specific additional constraints are added. The most widely used inverse method in DOT is a linear approach based on Minimum Norm Estimate (MNE) originally proposed for solving the inverse problem of MagnetoencephaloGraphy(MEG) and Electroencephalography (EEG) source localization^20^. It minimizes the $L_2$ norm of the reconstruction error along with Tikhonov regularization^9,12,14,21–23^. Other strategies to solve DOT inverse problem have also been considered, such as sparse regularization using the $L_1$ norm^23–27^ and Expectation Maximization (EM) algorithm^28^. A non-linear method based on hierarchical Bayesian model for which inference is obtained through an iterative process^29,30^ has been proposed and applied on finger tapping experiments^11^.

Maximum Entropy on the Mean (MEM) framework was first proposed by Amblard et al.^31^ and then applied and carefully evaluated by our group in the context of EEG/MEG source imaging^32,33^. The MEM framework was specifically designed and evaluated for its ability to recover spatially extended generators^34–37^. We recently demonstrated its excellent performances when recovering the spatial extent of the underlying generator in the context of focal sources^38^ and when applied on clinical epilepsy data^39,40^. In addition to its unique ability to recover the spatial extent of the underlying generators, we also demonstrated MEM’s excellent reconstruction spatial accuracy in low SNR conditions, with the ability to limit the influence of distant spurious sources^34,36,38,40–42^.

We believe that these important aspects should be carefully considered in the context of fNIRS reconstruction. The first one is the ability to accurately recover the spatial extent of the underlying hemodynamic activity for both focal and extended generators. The second one is to provide robust reconstruction results when data SNR decreases, especially when considering the fact that it is challenging to maintain a good intra-subject consistence using continuous-wave fNIRS due to its relatively low SNR^43^. Therefore, our main objective was to adapt the MEM framework for fNIRS reconstruction and carefully evaluate its performance. Moreover, fNIRS reconstruction results tends to be biased towards more superficial regions, because the light sensitivity profile decreases exponentially with the depth of the generators^44^. To overcome this bias, we implemented and evaluated a depth weighted variant of the MEM framework.

The article is organized as follows. The methodology of depth weighted MEM for DOT is first presented. Then, we described our validation framework using realistic simulations and associated validation metrics. fNIRS reconstruction using MEM was compared with widely used depth weighted Minimum Norm Estimate (MNE) inverse solution. Finally, illustrations of the methods on finger tapping fNIRS data set acquired with two different montages from 6 healthy subjects are provided and compared with functional Magnetic Resonance Imaging (fMRI) results.

## Material and methods

### Statement

All methods were carried out in accordance with relevant guidelines and regulations. All subjects have signed written informed consent forms for this study which was approved by the Central Committee of Research Ethics of the Minister of Health and Social Services Research Ethics Board, Québec, Canada.

### fNIRS reconstruction

To perform fNIRS reconstructions, the relationship between measured optical density changes on the scalp and wavelength specific absorption changes within head tissue is usually expressed using the following linear model^6^:

1 $$\begin{aligned} \begin{aligned} Y=AX+e \end{aligned} \end{aligned}$$

where *Y* is a matrix ($p\times t$) which represents the wavelength specific measurement of optical density changes in *p* channels at *t* time samples. *X* ($q\times t$) represents the unknown wavelength specific absorption changes in *q* locations along the cortex at time *t*. *A* ($p\times q$) is called the light sensitivity matrix which is actually the forward model relating absorption changes in the head to optical density changes measured in each channel. Finally, *e* ($p\times t$) models the additive measurement noise. Solving the fNIRS tomographic reconstruction problem consists in solving an inverse problem which can be seen as the estimation of matrix *X* (i.e. the amplitude for each location *q* at time *t*). However, this problem is ill-posed and admits an infinite number of possible solutions. Therefore, solving the DOT inverse problem requires adding additional prior information or regularization constraints to identify a unique solution.

In DOT studies, anatomical constraints can be considered by defining the reconstruction solution space (i.e. where *q* is located ) within the gray matter volume^45^ or along the cortical surface^46,47^. In EEG and MEG source localization studies^32,33,48^, it also is common to constrain the reconstruction along the cortical surface. In this study, the reconstruction space was considered as the mid surface defined as the middle layer between gray matter/pial and gray/white matter interfaces^49^.

### Minimum norm estimation (MNE)

Minimum norm estimation is one of the most widely used reconstruction methods in DOT^9,11–15,22^. Such estimation can be expressed using a Bayesian formulation which solves the inverse problem by estimating the posterior distribution $P(X|Y) = \frac{P(Y|X)P(X)}{P(Y)}$ (i.e. the probability distribution of parameter *X* conditioned on data *Y*). A solution can be computed by imposing Gaussian distribution priors on the generators *X* ($P(X) = N(0, {\Sigma }_s^{-1})$) and the noise *e* ($P(e) = N(0, {\Sigma }_d^{-1})$). $\Sigma _d$ is the inverse of the noise covariance which could be estimated from baseline recordings. $\Sigma _s$ is the inverse of the source covariance which is assumed to be an identity matrix in conventional MNE.

The Maximum a Posteriori (MAP) estimator of the posterior distribution *P*(*X*|*Y*) can be obtained using maximum likelihood estimation:

2 $$\begin{aligned} \begin{aligned} {\hat{X}}_{MNE}&= argmin\left( ||(Y-AX)||^2_{{\Sigma }_d} + \lambda ||X||^2_{{\Sigma }_s} \right) \\&= (A^T{\Sigma }_dA + {\lambda }{\Sigma }_s)^{-1}A^T{\Sigma }_dY \end{aligned} \end{aligned}$$

where ${{\widehat{X}}}_{MNE}$ is the reconstructed absorption changes along the cortical surface. $\lambda$ is a hyperparameter to regularize the inversion using the priori minimum norm constraint $\vert \vert X\vert \vert _{\Sigma _s}^{2}$. In this study, we applied the standard L-Curve method^50^ to estimate $\lambda$.

### Depth weighted MNE

Standard MNE solutions assumes $\Sigma _s =I$, which then tends to bias the inverse solution towards the generators exhibiting large sensitivity in the forward model, therefore the most superficial ones^51^. When compared to EEG-MEG source localization, such bias is even more pronounced in fNIRS since within the forward model light sensitivity values decrease exponentially with the depth^44^. This bias can be compensated by scaling the source covariance matrix such that the variances are equalized^51,52^. In the context of DOT, depth weighted MNE has been proposed by Culver et al.^53^ as an approach to compensate this effect and applied in different studies^9,12,14,15,22^. In practice, depth weighting can be formulated differently, here we consider a generalized expression for the implementation of depth weighted MNE as proposed in Lin et al.^54^. It consists in initializing the source covariance matrix as $\Sigma _s^{-1/2} = \Lambda$, resulting in a so called depth weighted MNE solution, described as follows:

3 $$\begin{aligned} \begin{aligned} {\hat{X}}_{dMNE}&= argmin\left( ||(Y-AX)||^2_{{\Sigma }_d} + \lambda ||X||^2_{{\Sigma }_s} \right) \\&= (A^T{\Sigma }_dA + {\lambda }(\Lambda \Lambda ^t)^{-1})^{-1}A^T{\Sigma }_dY\\ diag(\Lambda )&= \frac{1}{diag\left( (A^T {\Sigma }_d A)\right) ^{\omega }} \end{aligned} \end{aligned}$$

Depth weighted MNE solution takes into account the forward model *A* for each position in the brain and therefore penalizes most superficial regions exhibiting larger amplitude in *A*, by enhancing the contribution to deeper regions. $\omega$ is a weighting parameter tuning the amount of depth compensation to be applied. The larger is $\omega$, the more depth compensation is considered. $\omega =0$ would therefore refer to no depth compensation and an identity source covariance model. $\omega =0.5$ refers to standard depth weighting approach mentioned above. In the present study, we carefully evaluated the impact of this parameter on DOT accuracy with a set of $\omega$ values (i.e. $\omega = 0, 0.1, 0.3, 0.5, 0.7 \,and\, 0.9$).

### Maximum entropy on the mean (MEM) for fNIRS 3D reconstruction

#### MEM framework

The main contribution of this study is the first adaptation and evaluation of MEM method^31–33^ to perform DOT reconstructions in fNIRS. Within the MEM framework, the intensity of *x*, i.e. amplitude of *X* at each location *q* in Eq. (1), is considered as a random variable, described by the following probability distribution $dp(x) = p(x)dx$. The Kullback-Leibler divergence or $\nu$-entropy of *dp*(*x*) relative to a prior distribution $d\nu (x)$ is defined as,

4 $$\begin{aligned} S_{v}(dp(x)) = - \int _{x}log\left( \frac{dp(x)}{d\nu (x)}\right) dp(x) = - \int _{x} f(x)log(f(x))d\nu (x) \end{aligned}$$

where *f*(*x*) is the $\nu$-density of *dp*(*x*) defined as $dp(x)=f(x)d\nu (x)$. Following a Bayesian approach to introduce the data fit, we denote $C_{m}$ as the set of probability distributions on *x* that explains the data on average:

5 $$\begin{aligned} Y-[A|I_q]\begin{bmatrix} E_{dp}[x] \\ e \end{bmatrix} = 0,\qquad dp \in C_{m} \end{aligned}$$

where *Y* represents the measured optical density changes, $E_{dp}[x] = \int xdp(x)$ represents the statistical expectation of *x* under the probability distribution *dp*, and $I_q$ is an identity matrix of $(q\times q)$ dimension. Therefore, within the MEM framework, a unique solution of *dp*(*x*) could be obtained,

6 $$\begin{aligned} dp^{*}(x) = argmax_{dp(x)\in C_{m}}\left( S_{v}(dp(x))\right) \end{aligned}$$

The solution of $dp^{*}(x)$ can be solved by maximizing the $\nu$-entropy which is a convex function. It is equivalent to minimize an unconstrained concave Lagrangian function i.e., $L(dp(x),\kappa ,\lambda )$, along with two Lagrangian constraint parameters, i.e., $\kappa$ and $\lambda$. It is finally equivalent to maximize a cost function $D(\lambda )$ which is described as,

7 $$\begin{aligned} D(\lambda )\ =\lambda ^TY-F_v(A^T\lambda )-\frac{1}{2}\lambda ^T\mathrm {\Sigma }_d^{-1} {{(\mathrm {\Sigma }}_d^{-1})}^T\lambda \end{aligned}$$

where $\Sigma _d^{-1}$ is the noise covariance matrix. $F_v$ represents the free energy associated with reference $d\nu \left( x\right)$. It is important to mention that $D(\lambda )$ is now an optimization problem within a space of dimension equal to the number of sensors. Therefore, if we estimate $\lambda ^*=\ argmax_\lambda D(\lambda )$, the unique solution of MEM framework is then obtained from the gradient of the free energy.

8 $$\begin{aligned} {{\hat{X}}}_{MEM}\ =\ \mathrm {\nabla }_\xi F_\nu ^*(\xi )|_{\xi =A^T\lambda ^*} \end{aligned}$$

For further details on MEM implementation and theory we refer the reader to^31–33^.

#### Construction of the prior distribution for MEM estimation

To define the prior distribution $d\nu (x)$ mentioned above, we assumed that brain activity can be depicted by a set of $\text {K}$ non-overlapping and independent cortical parcels. Then the reference distribution $d\nu (x)$ can be modeled as,

9 $$\begin{aligned} d\nu (x) = \prod _{k=1}^{K}[(1-\alpha _k)\delta (x_{k}) + \alpha _kN(\mu _k,\Sigma _k)]dx_{k},\qquad 0<\alpha _k<1 \end{aligned}$$

Each cortical parcel k is characterized by an activation state, defined by the hidden variable $S_k$, describing if the parcel is active or not. Therefore we denote $\alpha _k$ as the probability of $k$th parcel to be active, i.e., $Prob(S_k = 1)$. $\delta _k$ is a Dirac function that allows to “switch off” the parcel when considered as inactive (i.e., $S_k = 0$). $N(\mu _k,\Sigma _k)$ is a Gaussian distribution, describing the distribution of absorptions changes within the $k$th parcel, when the parcel is considered as active ($S_k = 1$). This prior model, which is specific to our MEM inference, offers a unique opportunity to switch off some parcels of the model, resulting in accurate spatial reconstructions of the underlying activity patterns with their spatial extent, as carefully studied and compared with other Bayesian methods in Chowdhury et al.^33^.

The spatial clustering of the cortical surface into *K* non-overlapping parcel was obtained using a data driven parcellization (DDP) technique^55^. DDP consisted in first applying a projection method, the multivariate source prelocalization (MSP)^56^, estimating a probability like coefficient (MSP score) between 0 and 1 for each vertex of the cortical mesh, characterizing its contribution to the data. DDP is then obtained by using a region growing algorithm, along the tessellated cortical surface, starting from local MSP maxima. Once the parcellization is done, the prior distrubution $d\nu (x)$ is then a joint distribution expressed as the multiplication of individual distribution of each parcel in Eq. (9) assuming statistical independence between parcels,

10 $$\begin{aligned} d\nu (x) = d\nu _1(q_1)d\nu _2(q_2)...d\nu _k(q_k)...d\nu _K(q_K) \end{aligned}$$

where $d\nu (x)$ is the joint probability distribution of the prior, $d\nu _k(q_k)$ is the individual distribution of the parcel *k* described as Eq. (9).

To initialize the prior in Eq. (9), $\mu _k$ which is the mean of the Gaussian distribution, $N(\mu _k,\Sigma _k)$, was set to zero. $\Sigma _k$ at each time point *t*, i.e. $\Sigma _k(t)$, was defined by Eq. (11) according to Chowdhury et al.^33^,

11 $$\begin{aligned} \begin{aligned} \Sigma _k(t)&= \eta (t) W_k(\sigma )^TW_k(\sigma )\\ \eta (t)&= 0.05 \frac{1}{\mathscr {P}_{k}} \sum _{i\in \mathscr {P}_{k}}{\hat{X}}^2_{MNE}(i,t) \end{aligned} \end{aligned}$$

where $W_k(\sigma )$ is a spatial smoothness matrix, defined by Friston et al.^57^, which controls the local spatial smoothness within the parcel according to the geodesic surface neighborhood order. Same value of $\sigma = 0.6$ was used as in Chowdhury et al.^33^. $\eta (t)$ was defined as $5\%$ of the averaged energy of MNE solution within each parcel $\mathscr {P}_{k}$ at time t. Finally, we can substitute this initialization into Eq. (9) to construct the prior distribution $d\nu (x)$, and then obtain the MEM solution using Eq. (8).

It is worth mentioning that we did not use MNE solution as the prior of $\mu _k$ in Eq. (9) at all, which was actually initialized to 0 in our framework. We only used $5\%$ of the averaged energy of MNE solution, over the parcel *k*, to set the prior for covariance $\Sigma _k$. The posterior estimation of parameter $\mu _k$ was estimated from the Bayesian framework by conditioning with data. Moreover, the prior of MEM framework is a mixture of activation probability $\alpha _k$ and a Gaussian distribution [see Eq. (9)], in which the prior for $\alpha _k$ was informed by a spatio-temporal extension of the MSP score (see Chowdhury et al.^33^ for further details). These aspects completely differentiate MEM from approaches that iteratively update reconstruction results initialized by a MNE solution.

#### Depth weighted MEM

In addition to adapting MEM for fNIRS reconstruction, we also implemented for the first time, depth weighting within the MEM framework. Two depth weighting parameters, $\omega _1$ and $\omega _2$, were involved in this process. $\omega _1$ was used to apply depth weighting on the source covariance matrix $\Sigma _k$ of each parcel *k* in Eq. (11). $\omega _2$ was applied to solve the depth weighted MNE, as described in Eq. (3), before using those prior to initialize the source covariance model within each parcel of the MEM model. Therefore, the standard MNE solution ${\hat{X}}_{MNE}(i,t)$ in Eq. (11) was replaced by the depth weighted version of MNE solution ${\hat{X}}_{dMNE}(i,t)$ described by Eq. (3). Consequently, the depth weighted version of $\Sigma _k(t)$ is now defined as,

12 $$\begin{aligned} \begin{aligned} \Sigma _k(t)_{dw}&= \Lambda _{\mathscr {P}_{k}}\eta (t)_{dw}W_k(\sigma )^TW_k(\sigma )\\ \eta (t)_{dw}&= 0.05 \frac{1}{\mathscr {P}_{k}} \sum _{i\in \mathscr {P}_{k}}{\hat{X}}^2_{dMNE}(i,t) \end{aligned} \end{aligned}$$

where $\Lambda _{\mathscr {P}_{k}}$ is the depth weighting matrix for each pacel *k*, in which $\omega _1$ was involved to construct this scaling matrix as described in Eq. (3). This initialization followed the logic that depth weighting is in fact achieved by scaling the source covariance matrix. The other depth weighting parameter, $\omega _2$, was considered when solving ${\hat{X}}_{dMNE}(i,t)$, therefore avoiding biasing the initialization of the source covariance with a standard MNE solution.

To comprehensively compare MEM and MNE and also to investigate the behavior of depth weighting, we first evaluated the reconstruction performance of MNE with different $\omega _2$ (i.e. step of 0.1 from 0 to 0.9). Then two of these values (i.e. $\omega _2 = 0.3$ and 0.5) were selected for the comparison with MEM since they performed better than the others. Note that the following expressions of depth weighted MEM will be denoted as MEM($\omega _1$, $\omega _2$) to represent the different depth weighting strategies.

#### Accuracy of temporal dynamics

The last contribution of this study was to improve the temporal accuracy of MEM solutions. In classical MEM approach^33^, ${\hat{X}}_{MNE}(i,t)$ in Eq. (12) was globally normalized by ${\max \limits _{i \in \Omega , t \in T}({\hat{X}}_{MNE}(i,t))}$, where $\Omega$ represents all the possible locations along the cortical surface and *T* is the whole time segment. Therefore, the constructed prior along the time actually contained the temporal scaled dynamics from MNE solution. To remove this effect, we performed local normalization for ${\hat{X}}_{dMNE}(i,t)$ at each time instance *t*, i.e., by dividing by $\max \limits _{i \in \Omega }({\hat{X}}_{dMNE}(i,t))$. This new feature would preserve the spatial information provided by prior distribution, while allowing MEM to estimate the temporal dynamics only from the data.

### Validation of fNIRS reconstruction methods

We evaluated the performance of the two fNIRS reconstruction methods (i.e., MEM and MNE), first within a fully controlled environment involving the use of realistic simulations of fNIRS data using montage 1, followed by evaluations on real data acquired with a well controlled finger tapping paradigm using montage 2. One subject was involved in acquisitions using montage 1 and five subjects participated in acquisitions using montage 2.

**Figure 1:**
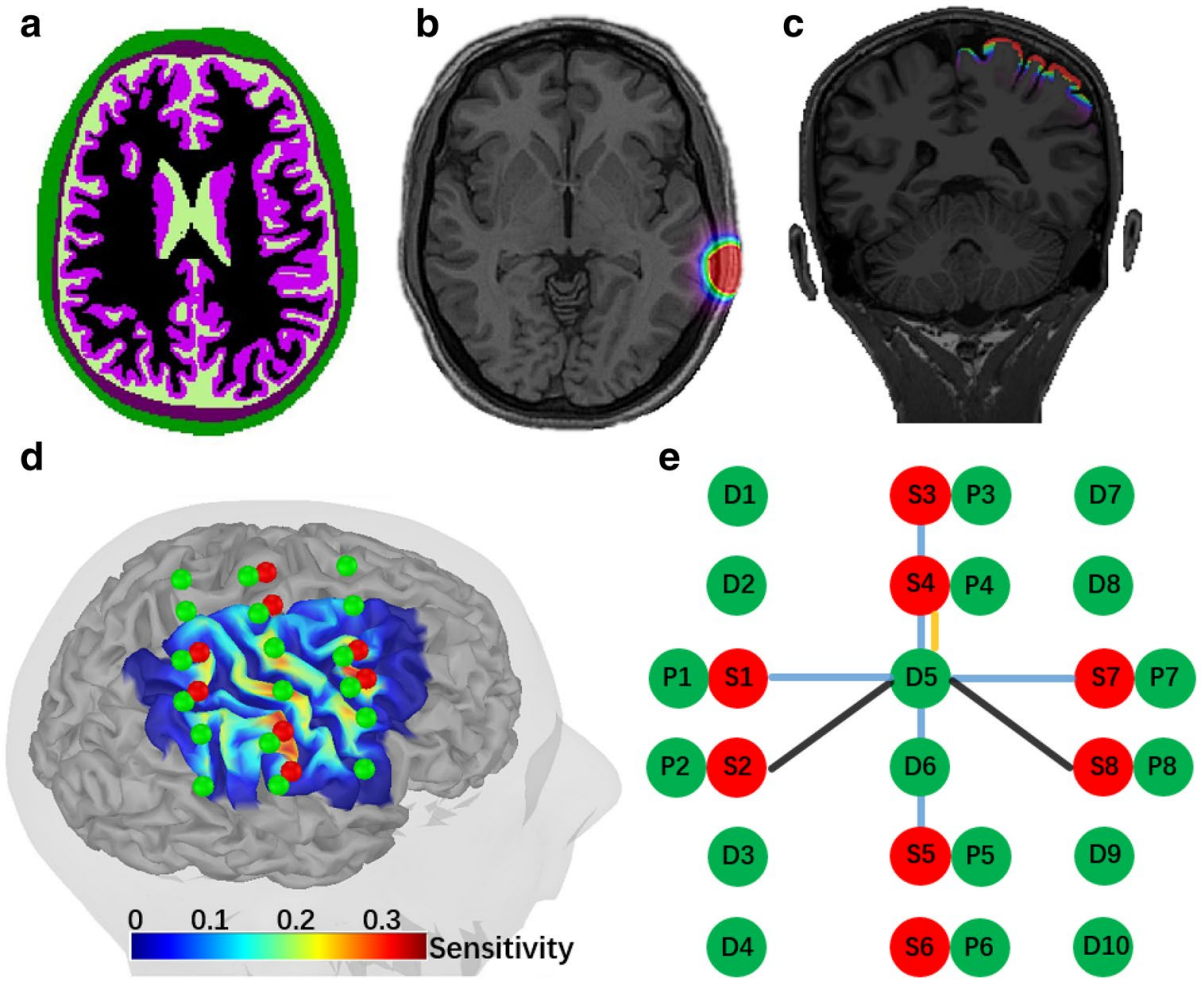
fNIRS measurement montage 1 and the anatomical model considered for DOT forward model estimation. (**a**) Anatomical 3D MRI segmented in five tissues, namely, scalp (green), skull (brown), CSF (light green), gray matter (purple) and white matter (black). (**b**) Optical fluence of one optode calculated through Monte Carlo simulation of Photons within this head model, using MCXLab. (**c**) Sensitivity profile of the whole montage in volume space. (**d**) Sensitivity profile, i.e. the summation of sensitivity map of all channels, along the cortical surface. Green dots represent detectors, including one proximity detector $0.7\,\text {cm}$ for each source, and red dots represent sources. (**e**) double-density montage 1 considered for this acquisition. There were 50 channels in total, 12 of $3.8\,\text {cm}$ (black), 24 of $3\,\text {cm}$ (blue), 6 of $1.5\,\text {cm}$ (yellow) and 8 of close distance (0.7 cm) channels. Figure created by Brainstorm^59^ using the NIRSTORM plugin developed by our team (https://github.com/Nirstorm).

*Montage 1* A full Double Density (DD) montage (see Fig. 1) which is a widely used fNIRS montage, was considered given that it allows sufficient dense spatial coverage of fNIRS channel to allow local DOT^58^. One healthy subject (20 years old, right handed) underwent fNIRS acquisitions with this DD montage, involving the two following sessions,

- A 10 minutes resting state session was acquired to add realistic physiology noise to corrupt our noise-free simulations, thus generating highly realistic fNIRS simulations. These resting-state fNIRS data captured spontaneous fluctuations in fNIRS signals that are related to intrinsic brain activity as well as the physiological noise of non-cerebral origin, associated with systemic blood circulations^1^. The subject was seating on a comfortable armchair and instructed to keep the eyes open and to remain awake. The optodes of the full DD montage (i.e. 8 sources and 10 detectors resulting in 50 fNIRS channels) are presented in Fig. 1e. The montage composed of 6 second-order distance channels (1.5 cm), 24 third-order channels(3 cm) and 12 fourth-order channels with 3.35 cm distance. In addition, we also added one proximity detector paired for each source to construct close distance channels (0.7 cm) in order to measure superficial signals within extra-cerebral tissues. To place the montage with respect to the region of interest, the center of the montage was aligned with the center of the right “hand knob” area, which controls the left hand movement^60^, projected on the scalp surface and then each optodes were projected on the scalp surface (see Fig. 1d).
- The subject was asked to sequentially tap the left thumb against the other digits around 2Hz, therefore the main elicited hemodynamic response was indeed expected over the right hand knob area. The finger tapping paradigm consisted in 10 blocks of 30s tapping task and each of them was followed by a 30 to 35s resting period. The beginning/end of each block was informed by an auditory cue.

*Montage 2*: Five subjects underwent fNIRS acquisitions with personalized optimal montage^19^ during a similar aforementioned finger tapping task. Personalized optimal montage was applied to maximize the fNIRS sensitivity to the hand knob within right primary motor cortex of each participant. Please find further details in Supplementary material S4.

#### MRI and fMRI Data acquisitions

Anatomical MRI data were acquired on those 6 healthy subjects ($25\pm 6$ years old, right-handed) and were considered to generate realistic anatomical head models. MRI data were acquired in a GE 3T scanner at the PERFORM Center of Concordia University, Montréal, Canada. T1-weighted anatomical images were acquired using the 3D BRAVO sequence ($1\times 1\times 1\,{\text {mm}}^{3}$, 192 axial slices, $256\times 256$ matrix), whereas T2-weighted anatomical images were acquired using the 3D Cube T2 sequence ($1\times 1\times 1\,{\text {mm}}^{3}$ voxels, 168 sagittal slices, $256\times 256$ matrix).

Participants also underwent functional MRI acquisition while performing the same finger opposition tasks considered in fNIRS. fNIRS and fMRI data were acquired in two different sessions, one week apart from each other. fMRI acquisition consisted in a gradient echo EPI sequence ($3.7\times 3.7\times 3.7\,{\text {mm}}^{3}$ voxels, 32 axial slices, $\text {TE}=25\,\text {ms}$, $\text {TR}=2000\,\text {ms}$). fMRI Z-maps were generated by standard first-level fMRI generalize linear model analysis using FEAT from FSL v6.0.0 software^61,62^.

#### fNIRS data acquisition

fNIRS acquisitions were conducted at the PERFORM Center of Concordia University using a Brainsight fNIRS device (Rogue Research Inc., Montréal, Canada), equipped with 16 dual wavelength sources (685*nm* and 830*nm*), 32 detectors and 16 proximity detectors (for short distance channels). All montages (i.e., double density and optimal montages) were installed to cover the right motor cortex. Knowing a priori the exact positions of fNIRS channels estimated on the anatomical MRI of each participant, we then used a 3D neuronavigation system (Brainsight TMS navigation system, Rogue Research Inc.) to guide the installation of the sensors on the scalp. This neuronavigation system provided real-time feedback of the optodes targeted positions, while installing them on the subject’s head. Finally, every sensor was glued on the scalp using a clinical adhesive, collodion, to prevent motion and ensure good contact to the scalp^19,63^. For further details about this personalized installation procedure, please refer to our previous publication^19^.

#### fNIRS forward model estimation

T1 and T2 weighted anatomical images were processed using FreeSurfer V6.0^49^ and Brain Extraction Tool2 (BET2)^61^ in FMRIB Software Library (FSL) to segment the head into 5 tissues (i.e. scalp, skull, Cerebrospinal fluid (CSF), gray matter and white matter see Fig. 1a).

Same optical coefficients used in^19,63^ for the two wavelengths considered during our fNIRS acquisition, 685*nm* and 830*nm*, were assigned to each tissue type mentioned above. Fluences of light for each optode (see Fig. 11b) was estimated by Monte Carlo simulations with $10^8$ photons using MCXLAB developed by Fang and Boas^64^ and Yu et al.^65^ (http://mcx.space/). Sensitivity values were then computed using the adjoint formulation and were normalized by the Rytov approximation^6^.

For each source-detector pair of our montages, the corresponding light sensitivity map was first estimated in a volume space, and then further constrained to the 3D mask of gray matter tissue (see Fig. 1c), as suggested by Boas and Dale^45^. Then, these sensitivity values within the gray matter volume were projected along the cortical surface (see Figs. 1d and S4c) using the Voronoi based method proposed by^32^. We considered the mid-surface from FreeSurfer as the cortical surface. This surface was downsampled to 25, 000 vertices. This volume to surface interpolation method has the ability to preserve sulco-gyral morphology^32^. After the interpolation, the sensitivity value of each vertex of the surface mesh represents the mean sensitivity of the corresponding volumetric Voronoi cell (i.e., a set of voxels that have closest distances to a certain vertex than to all other vertices).

#### fNIRS data preprocessing

Using the coefficient of variation of the fNIRS data, channels exhibiting a standard deviation larger than $8\%$ of the signal mean were rejected^14,66–68^. Superficial physiological fluctuations were regressed out at each channel using the average of all proximity channels’ (0.7 cm) signals^12^. All channels were then band-pass filtered between 0.01 Hz and 0.1 Hz using a 3rd order Butterworth filter. Changes in optical density (i.e., $\Delta OD$) were calculated using the conversion to log-ratio. Finally, $\Delta OD$ of finger tapping data were block averaged around the task onsets. Note that since sensors were glued with collodion, we observed very minimal motion during the acquisitions. Real background signal considered to generate realistic simulations also underwent the same preprocessing.

#### Realistic simulations of fNIRS data

We first considered realistic simulations of fNIRS data to evaluate DOT methods within a fully controlled environment. To do so, theoretical task-induced HbO/HbR concentration changes were simulated within cortical surface regions with a variety of locations, areas and depths. Corresponding optical density changes in the channel space were then computed by applying the corresponding fNIRS forward model, before corrupting noise-free simulation using real resting state fNIRS baseline signal, allowing to add physiological fNIRS signal of cerebral and non-cerebral origin at different signal to noise ratio (SNR) levels.

**Figure 2:**
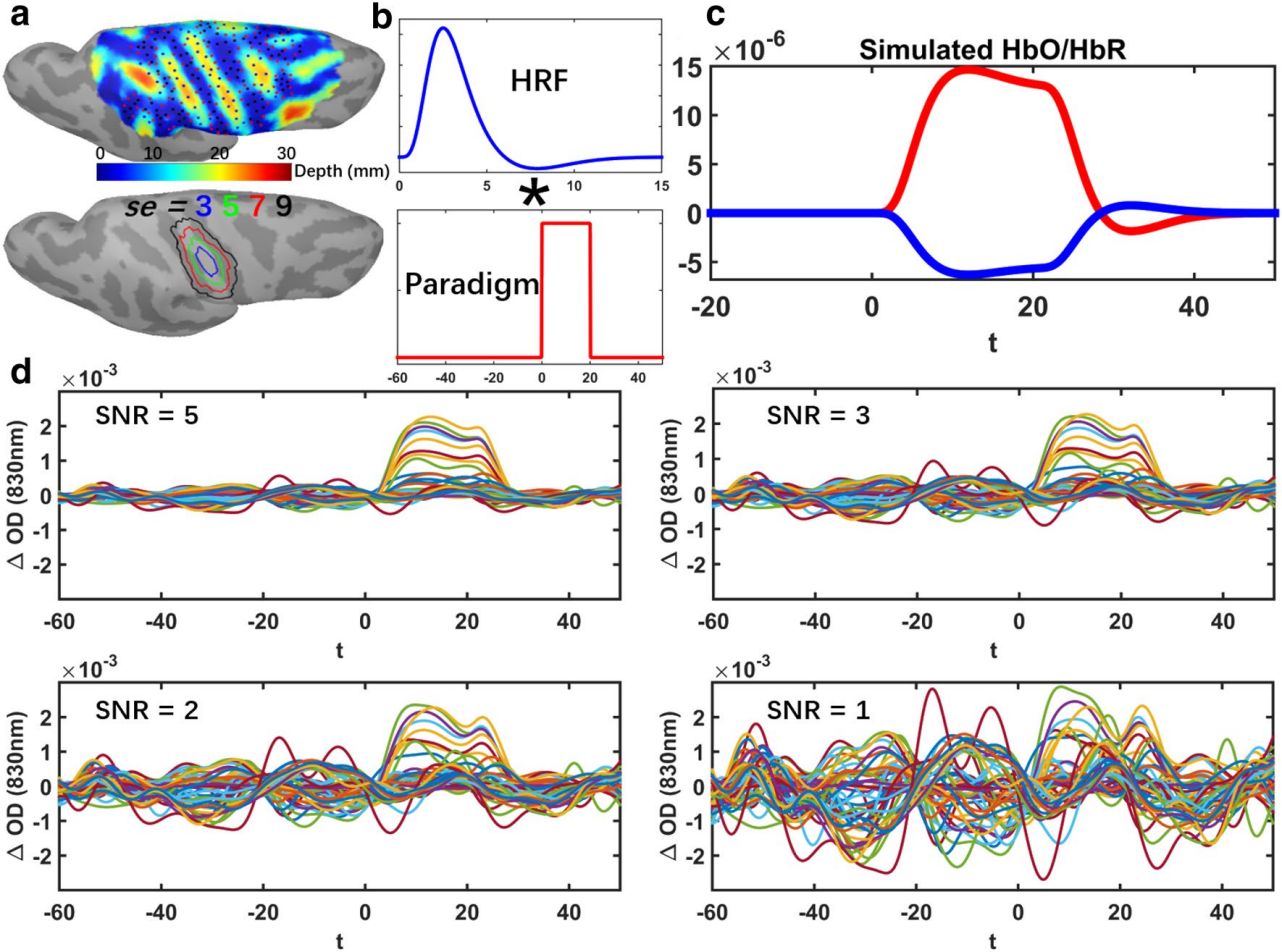
Workflow describing our proposed realistic fNIRS simulation framework. (**a**) 100 Superficial seeds (black dots), 100 Middle seeds (red dots), 50 Deep seeds (blue dots) with spatial extent of $Se = 3,5,7,9$ neighbourhood order within the field of view. (**b**) Convolution of a canonical HRF model with an experimental block paradigm (60*s* before and 50*s* after the onset). (**c**) Simulated theoretical HbO/HbR fluctuations along the cortical surface within the corresponding generator. (**d**) Realistic simulations obtained by applying the fNIRS forward model and addition of the average of 10 trials of real fNIRS background measurements at 830 nm. Time course of $\Delta OD$ of all channels with SNR of 5, 3, 2 and 1 respectively are presented. Figure created by Brainstorm^59^ using the NIRSTORM plugin developed by our team (https://github.com/Nirstorm).

As presented in Fig. 2a, we defined three sets of evenly distributed seeds within the field of view of DOT reconstruction. The locations were selected with respect to the depth relative to the skull, namely we simulated 100 “superficial seeds”, 100 “middle seeds” and 50 “deep seeds”. The cortical regions in which we simulated an hemodynamic response were generated by region growing around those seeds, along the cortical surface. To simulate generators with different spatial extents (denoted here as Se), we considered four levels of neighborhood orders, growing geodesically along the cortical surface, resulting in spatial extents ranging from $\text {Se} = 3,5,7,9$ (corresponding areas of 3 to 40 $\text {cm}^2$). For simplification, these cortical regions within which an hemodynamic response was simulated will be denoted as “generators” in this paper. For each vertex within a “generator”, a canonical Hemodynamic Response Function (HRF) was convoluted with a simulated experimental paradigm which consisted in one block of 20*s* task surrounded by 60*s* pre-/post- baseline period (Fig. 2b). Simulated HbO/HbR fluctuations within the theoretical generator (Fig. 2c) were then converted to the corresponding absorption changes of two wavelengths (i.e., 685*nm* and 830*nm*). After applying the forward model matrix A in Eq. (1), we estimated the simulated, noise free, task induced $\Delta OD$ in all channels. Such a simulation procedure provided a fully controlled access to ground truth since the location and size of the generator along the cortical surface and the corresponding simulated hemodynamic response time courses (HbO/HbR) within each generator were fully known. Therefore, this controlled ground truth, defined in space along the cortical surface and along time, was then considered for quantitative validations of fNIRS reconstructions, when assessing localization error, spatial extent accuracy and accuracy of temporal reconstructions.

$\Delta OD$ of real resting state data were then considered as baseline data and used to add realistic fluctuations (noise) to these simulated noise-free signals. Over the 10min of recording, we randomly selected 10 baseline epochs of 120*s* each, free from any motion artifact by visual inspection. To mimic a standard fNIRS block average response, realistic simulations were obtained by adding the average of these 10 real baseline epochs to the theoretical noise-free simulated $\Delta OD$, at five SNR levels (i.e. $\text {SNR} = 5, 3, 2, 1$). SNR was calculated through the following equation,

13 $$\begin{aligned} SNR_{\lambda } = \frac{\max (abs(\Delta OD_{\lambda }[0,t_1]))}{mean(std(\Delta OD_{\lambda }[-t_0,0]))} \end{aligned}$$

where $\Delta OD_{\lambda }[0,t_1]$ is the optical density changes of a certain wavelength $\lambda$ in all channels during the period from 0*s* to $t_1 = 60s$. $std(\Delta OD_{\lambda }[-t_0,0])$ is the standard deviation of $\Delta OD_{\lambda }$ during baseline period along all channels. Simulated trials for each of four different SNR levels are illustrated in Fig. 2d. A total number of 4000 realistic simulations were considered for this evaluation study, i.e., $250\,\text {(seeds)}\times 4\,\text {(spatial extents)}\times 4\,\text {(SNR levels)}$. Note that resting state fNIRS baseline signal was preprocessed before adding to the simulated signals.

#### Validation metric

Following the previously described validation metrics^32,33,36,38^, we applied 4 quantitative metrics to access the spatial and temporal accuracy of fNIRS 3D reconstructions. Further details on the computation of those four validation metrics are reported in Supplementary material S1.

- **Area Under the Receiver Operating Characteristic (ROC) curve (AUC)** was used to assess general reconstruction accuracy considering both sensitivity and specificity. AUC score was estimated as the area under the ROC curve, which was obtained by plotting sensitivity as a function of (1- specificity). AUC ranges from 0 to 1, the higher it is the more accurate the reconstruction is.
- **Minimum geodesic distance (Dmin)** measuring the geodesic distance in millimeters, following the circumvolutions of the cortical surface, from the vertex that exhibited maximum of reconstructed activity to the border of the ground truth. Low Dmin values indicate better accuracy in estimating the location of the generator.
- **Spatial Dispersion (SD)** assessed the spatial spread of the estimated generator distribution and the localization error. It is expressed in millimeters. A reconstructed map with either large spatial spread around the ground truth or large localization error would result in large SD values.
- **Shape error(SE)** evaluated the temporal accuracy of the reconstruction. It was calculated as the root mean square of the difference between the normalized reconstructed time course and the normalized ground truth time course. Low SE values indicate high temporal accuracy of the reconstruction.

### Statistics

Throughout all of the quantitative evaluations among different methods involving different depth weighting factors $\omega$ in the results section, Wilcoxon signed rank test was applied to test the significance of the paired differences between each comparison. For each statistical test, we reported the median value of paired differences, together with its p-value (Bonferroni corrected). We are only showing results at 830nm for simulations, since the ones from 690nm under the same SNR level would have provided similar reconstructed spatiotemporal maps except for the reversed amplitudes. However, reconstruction results on real data indeed involved both wavelengths.

## Results

### Evaluation of MEM versus MNE using realistic simulations

We first investigated the effects of depth weighting factor ${boldsymbol{\omega }}_2$ selection for depth weighted MNE. To do so, we evaluated spatial and temporal performances of DOT reconstruction for a set of $\omega _2$ (step of 0.1 from 0 to 0.9). Based on those results reported in the Supplementary material S2 and Fig. S1, we decided to considered that most accurate fNIRS reconstructions were obtained when considering $\omega _2 = 0.3$ and 0.5 for depth weighted MNE. Therefore only those two values were further considered for comparison with MEM reconstructions.

**Figure 3:**
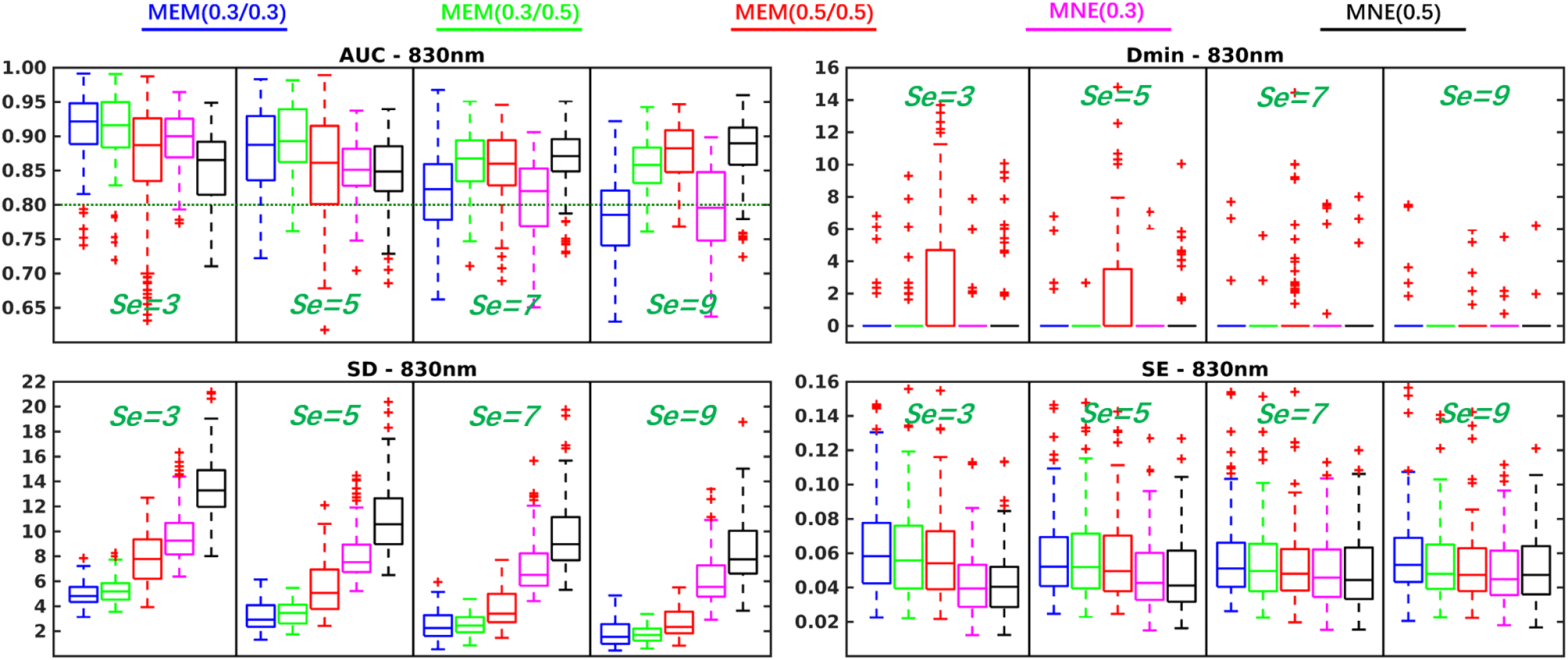
Evaluation of the performances of MEM and MNE using realistic simulations involving superficial seeds for different spatial extent ($Se = 3,5,7,9$). Boxplot representation of the distribution of four validation metrics for three depth weighted strategies of MEM and two depth weighted strategies of MNE, namely: MEM(0.3, 0.3) in blue, MEM(0.3, 0.5) in green, MEM(0.5, 0.5) in red, MNE(0.3) in magenta and MNE(0.5) in black. Results were obtained after DOT reconstruction of 830*nm* $\Delta OD$. Figure created by MATLAB version (R2016a) https://www.mathworks.com/products/matlab.html.

Comparison of the performance of MEM and MNE on superficial realistic simulations are presented in Table 1 and Fig. 3, for 4 levels of spatial extent ($Se = 3,5,7,9$), using boxplot distribution of the 4 validation metrics. We evaluated 3 depth weighted implementations of MEM, namely, MEM($\omega _1=0.3,\omega _2=0.3$), MEM(0.3, 0.5) and MEM(0.5, 0.5), as well as 2 depth weighted implementations of MNE, namely, MNE(0.3) and MNE(0.5).

For spatial accuracy, results evaluated using Dmin, we obtained median Dmin values of 0 mm for all methods, indicating the peak of the reconstructed map, was indeed accurately localized inside the simulated generator. It is worth mentioning that MEM(0.5, 0.5) provided few Dmin values larger than 0 mm in $Se=3$ and $Se=5$ cases, which consisted of superficial and focal generators. Since MEM accurately estimated the spatial extent, more depth weighting considered for MEM(0.5, 0.5) could results in focal and deeper reconstruction, hence resulting in non-zero Dmin values. On the other hand, MNE would over-estimate the size of the underlying generators, therefore resulting in 0 mm Dmin, but larger SD values in similar conditions.

When considering the general reconstruction accuracy using AUC, for focal generators such as $Se=3$ and 5, we found significant larger AUC (see Table 1) for MEM(0.3, 0.3) and MEM(0.3, 0.5) when compared to the most accurate version of MNE, i.e., MNE(0.3). When considering more extended generators, i.e., $Se=7$ and 9, MEM(0.3, 0.5) and MEM(0.5, 0.5) achieved significantly larger AUC than MNE(0.3). However, the AUC of MNE(0.5) was significantly larger than MEM(0.3, 0.3) for $Se=7$ as well as significantly larger than MEM(0.3, 0.5) and MEM(0.5, 0.5) for $Se=9$.

In terms of spatial extent of the estimated generator distribution and the localization error, MEM provided significantly smaller SD values among all the comparisons. Finally, for temporal accuracy of the reconstruction represented by SE, MNE provided significantly lower values, but with a small difference (e.g., 0.01 or 0.02, see results on real data as a reference of this effect size), than MEM among all comparisons when $Se=3, 5$.

Similar comparison between MEM and MNE were conducted respectively for middle seed simulated generators and deep seed simulated generators. Results were overall reporting similar trends when comparing MEM and MNE methods for middle and deep seeds, and as expected more depth weighting resulted in more accurate reconstructions (described in detail in supplementary material, Fig. S2 and Table S1for middle seeds, Fig. S3 and Table S2 for deep seeds).

**Table 1:**
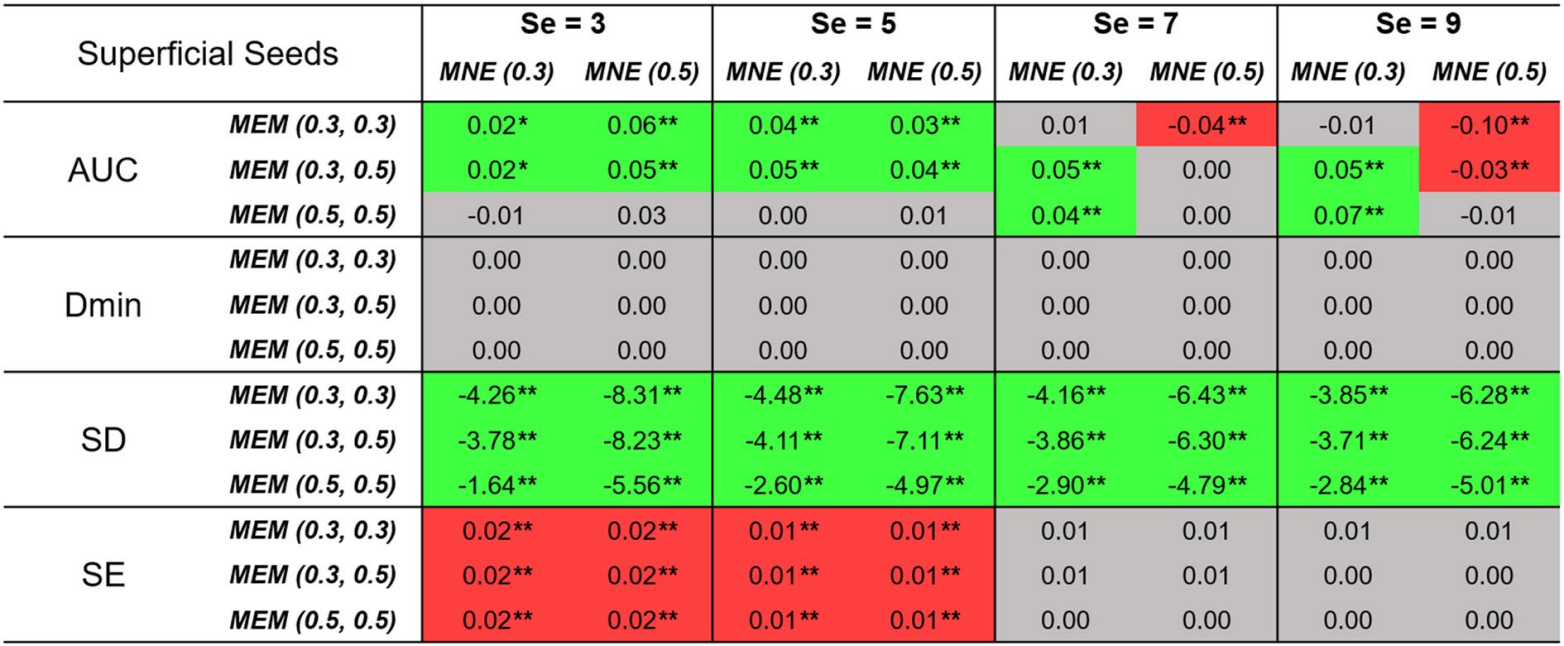
Wilcoxon signed rank test results of reconstruction performance comparison of MEM and MNE in superficial seeds case. Median values of paired difference are presented in the table.

*p* values were corrected for multiple comparisons using Bonferroni correction, * indicates $p<0.01$ and ** represents $p<0.001$. Median of the paired difference of each validation metrics is color coded as follows: green: MEM is significantly better than MNE, red: MNE is significantly better than MEM and gray: non-significance.

**Figure 4:**
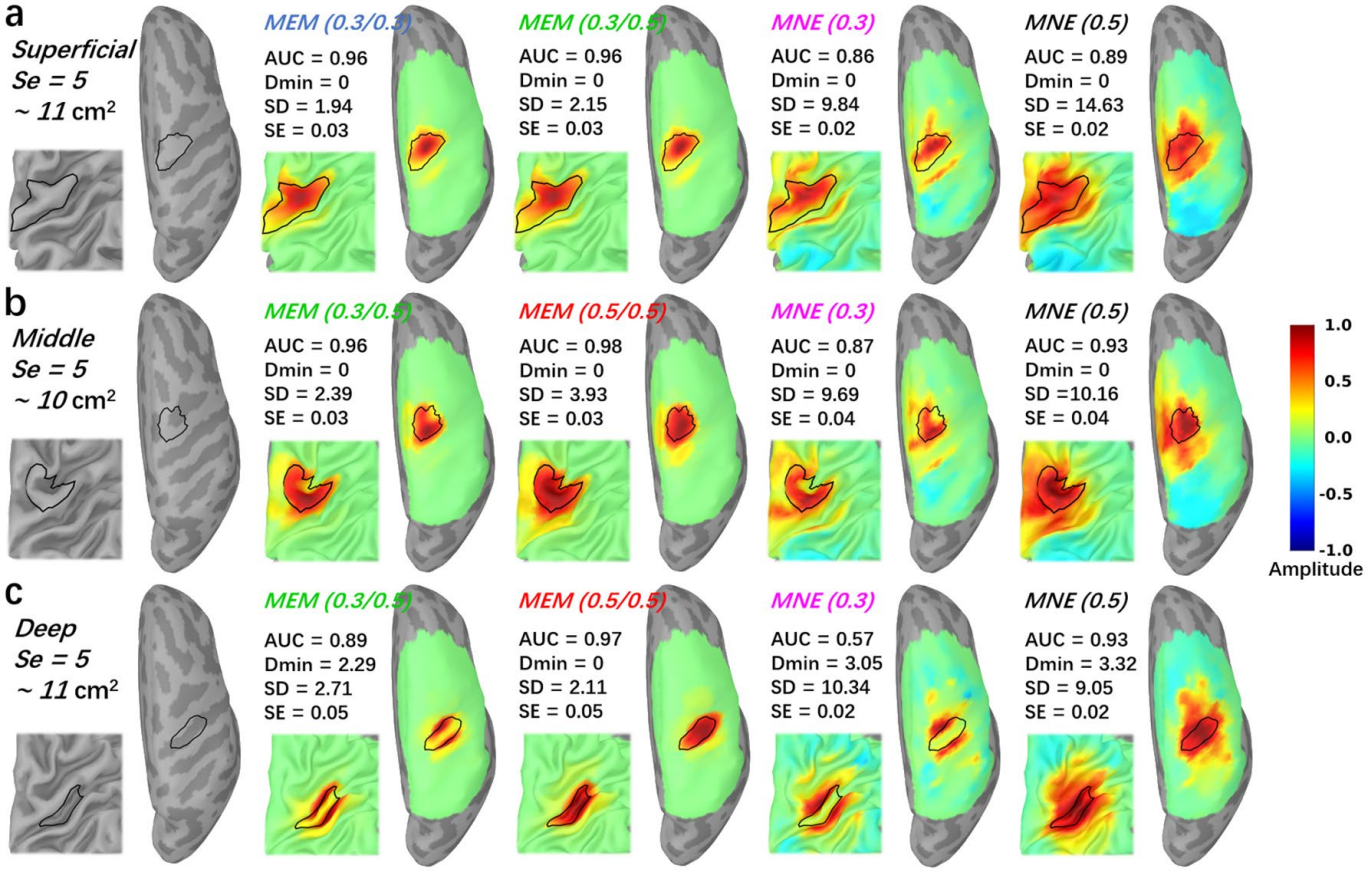
Comparisons of the reconstruction maps using MEM and MNE in realistic simulations. Three theoretical regions with spatial extent $Se= 5$ ($~11\, {\text cm}^2$) were selected near the ‘hand knob’ at different depth. The first column presents the locations and the size of the generator along the cortical surface. (**a**) Superficial seed case with reconstructed maps reconstructed using all MEM and MNE implementations considered in this study. (**b**) Middle seed case with reconstructed maps reconstructed using all MEM and MNE implementations considered in this study. (**c**) Deep seed case with reconstructed maps reconstructed using all MEM and MNE implementations considered in this study. $20\%$ inflated and zoomed maps are presented on the left corner of each figure. $100\%$ inflated right hemisphere are presented on the right side. All the maps were normalized by their own global maximum and no threshold was applied. Figure created by Brainstorm^59^ using the NIRSTORM plugin developed by our team (https://github.com/Nirstorm).

To further illustrate the performance of MEM and MNE as a function of the depth of the generator, we are presenting some reconstruction results in Fig. 4. Three generators with a spatial extent of $Se = 5$, were selected for this illustration. They were all located around the right “hand knob” area, and were generated from a superficial, middle and deep seed respectively. The first column in Fig. 4 shows the location and the size of the simulated generator, considered as our ground truth. The generator constructed from the superficial seed only covered the corresponding gyrus, whereas the generators constructed from the middle seed, included parts of the sulcus and the gyrus. Finally, when considering the deep seed, the simulated generator covered both walls of the sulcus, extended just a little on both gyri. For superficial case, MEM(0.3, 0.3) and MEM(0.3, 0.5) provided similar performances in term of visual evaluation of the results and quantitative evaluations ($AUC=0.96$, $Dmin=0\, {\text {mm}}$, $SD=1.94 \, {\text {mm}}, 2.15 \, {\text {mm}}$, $SE=0.03$). On the other hand, for the same simulations, MNE(0.3) and MNE(0.5) resulted in less accurate reconstructions, spreading too much around the true generator, as confirmed by validation metric, exhibiting notably large SD values ($AUC=0.86,0.89$, ${\text Dmin}=0 \, {\text {mm}}$, $SD=9.84 \, {\text {mm}}, 14.63 \, {\text {mm}}$, $SE=0.02$). When considering the simulation obtained with the middle seed, MEM(0.3, 0.5) retrieved accurately the gyrus part of the generator but missed the sulcus component, since less depth compensation was considered. When increasing depth sensitivity, MEM(0.5, 0.5) clearly outperformed all other methods, by retrieving both the gyrus and sulcus aspects of the generator, resulting in the largest $AUC=0.98$ and the lowest $SD=2.93 \, {\text {mm}}$. MNE(0.3) was not able to recover the deepest aspects of the generator, but also exhibited a large spread outside the ground truth area as suggested by a large $SD=9.69 \, {\text {mm}}$. MNE(0.5) was able to retrieve the main generator, but also exhibited a large spatial spread of $SD=10.16 \, {\text {mm}}$. When considering the generators obtained from the deep seed, MNE(0.3) only reconstructed part of gyrus, missing completely the main sulcus aspect of the generator, resulting in low AUC of 0.57 and large SD of 10.34 mm . MEM(0.3, 0.5) was not able to recover the deepest aspects of the sulcus, but reconstructed accurately the sulci walls, resulting in an AUC of 0.89 and a SD of 2.71 mm . MEM(0.5, 0.5) recovered the deep simulated generator very accurately, as demonstrated by the excellent scores ($AUC = 0.97$, $SD = 2.11 \, {\text {mm}}$) when compared to MNE(0.5). For those three simulations, all methods recovered the underlying time course of the activity with similar accuracy (i.e., similar SE values). In supplementary material, we added Video.1, illustrating the behavior of all the simulations and all methods, following the same layout provided in Fig. 4.

Note that for this quantitative evaluation of fNIRS reconstruction methods using realistic simulation framework, we considered fNIRS data at only one wavelength (830*nm*). Using single wavelength in the context simulation based evaluation is a common procedure in DOT literature^9,13,23,25,29,30,69^, since we may expect overall similar performances for 685 nm wavelength under the same SNR level.

### Effects of depth weighting on the reconstructed generator as a function of the depth and size of the simulated generators

**Figure 5:**
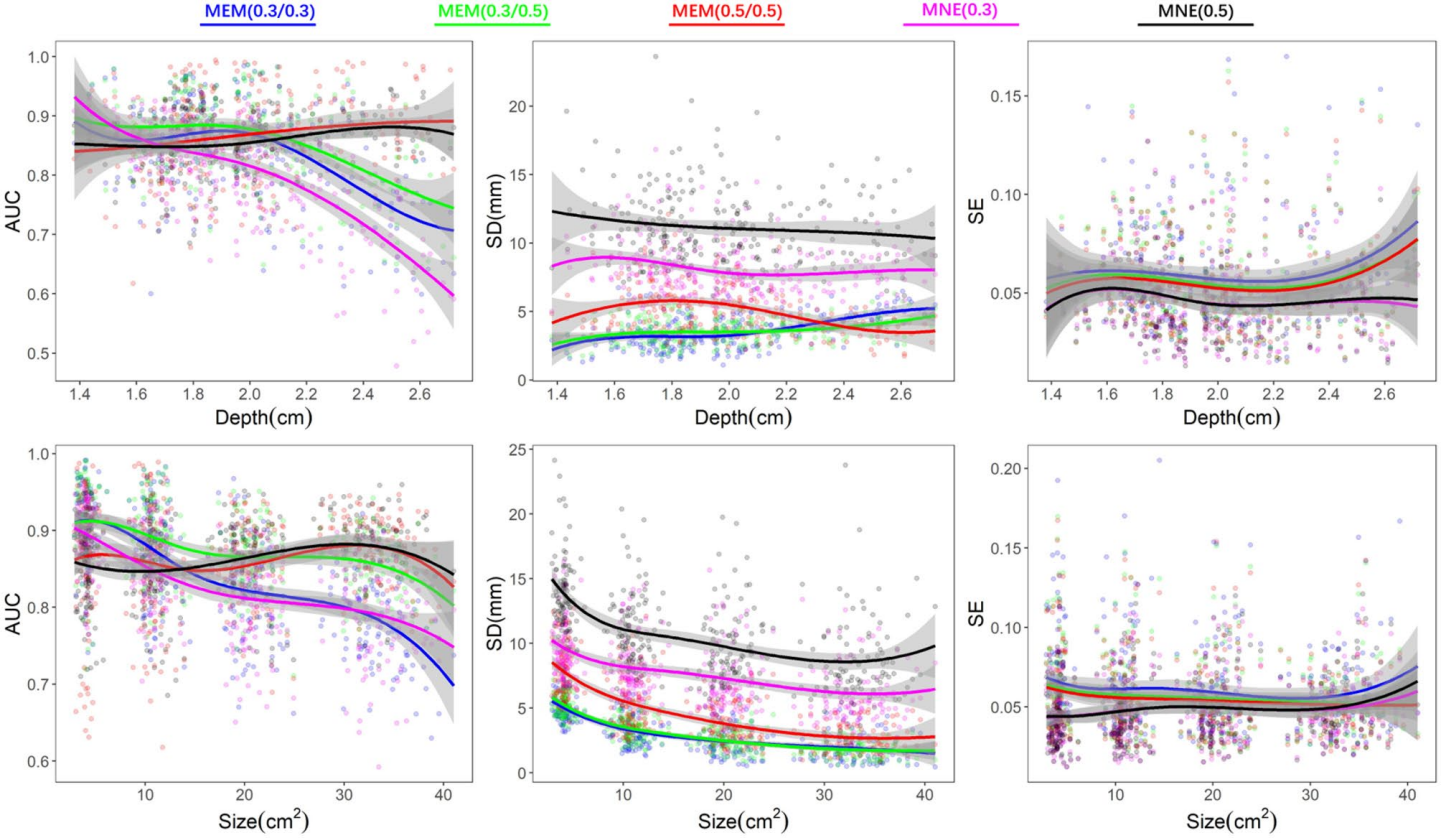
Effects of depth weighting on the depth and size of the simulated generators. First row demonstrates the validation matrices, AUC, SD and SE, as a function of depth of generators. We selected 250 generators created from all 250 seeds with a spatial extent of $SD=5$. Depth was calculated by the average of minimum Euclidean distance from each vertex, within each generator, to the head surface. Second row demonstrates the validation matrices, AUC, SD and SE, as a function of size of generators. Involving 400 generators which constructed from 100 superficial seeds with 4 different spatial extend of $Se = 3,5,7,9$. Line fittings were performed via a 4 knots spline function to estimate the smoothed trend and the shade areas represent $95\%$ confident interval. Color coded points represent the values of validation matrices of all involved generators. Figure created by MATLAB version (R2016a) https://www.mathworks.com/products/matlab.html.

To summarize the effects of depth weighting in 3D fNIRS reconstructions, we further investigated the validation metrics, AUC, SD and SE, as a function of depth and size of the simulated generators. Dmin was not included due to the fact that we did not find clear differences among methods throughout all simulation parameters from previous results. In the top row of Fig. 5, 250 generators created from all 250 seeds with a spatial extent of $Se=5$ were selected to demonstrate the performance of different versions of depth weighting as a function of the average depth of the generator. Whereas in the bottom row of Fig. 5, we considered 400 generators constructed from all 100 superficial seeds with 4 different spatial extents of $Se = 3,5,7,9$, to illustrate the performance of different versions of depth weighting as a function of the size of the generator. According to AUC, depth weighting was indeed necessary for all methods when the generator moved to deeper regions ($>2\, {\text{cm}}$) as well as when the size was larger than $20\, {\text{cm}}^2$. Moreover, any version of MEM always exhibited clearly less false positives, as indicated by lower SD values, than all of MNE versions, whatever was the depth or the size of the underlying generator. We found no clear trend and difference of temporal accuracy among methods when reconstructing generators of different depths and sizes.

### Robustness of 3D reconstructions to the noise level

**Figure 6:**
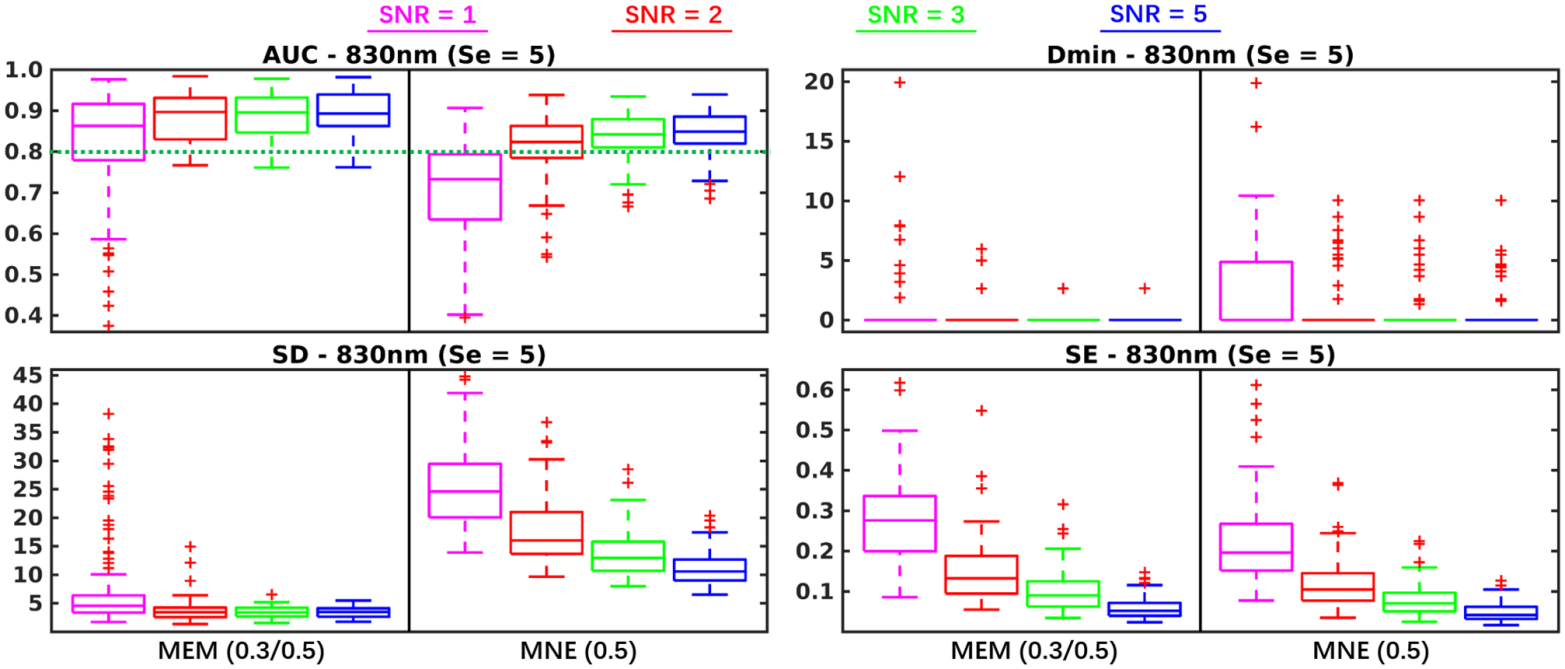
Evaluation of the performances of MEM and MNE at four different SNR levels. Boxplot representation of the distribution of four validation metrics for MEM(0.3, 0.5) and MNE(0.5) involving superficial seeds with spatial extent $Se=5$. SNR levels ($SNR=1,2,3,5$) are represented using different colors. Figure created by MATLAB version (R2016a) https://www.mathworks.com/products/matlab.html.

All previous investigations were obtained from simulations obtained with a SNR of 5, in this section we compared the effect of the SNR level in Fig. 6, on depth weighted versions of MNE and MEM, for superficial seeds only and generators of spatial extent $Se = 5$. We only compared MEM(0.3, 0.5) and MNE(0.5) considering the observation from previous results that these two methods were overall exhibiting best performances in this condition. Regarding Dmin, paired differences were not significant but MNE exhibited more Dmin values above 0 mm than MEM at all SNR levels, suggesting that MNE often missed the main generators while MEM was more accurate in reconstructing the maximum of activity within the simulated generator. Regarding AUC, MEM(0.3, 0.5) exhibited values higher than 0.8 at all SNR levels, whereas MNE(0.5) failed to recover accurately the generator for $SNR=1$. Besides, in Table 2, we found that difference of AUC between MEM and MNE increased when SNR level decreased, suggesting the good robustness of MEM when decreasing the SNR level. The difference of SD also increased when SNR levels decreased. Indeed, MEM exhibited stable SD values among most SNR levels (except $SNR =1$), whereas for MNE SD values were highly influenced by the SNR level. Finally, for both methods, decreasing SNR levels resulted in less accurate time course estimation (SE increased), slightly more for MEM when compared to MNE.

**Table 2:**
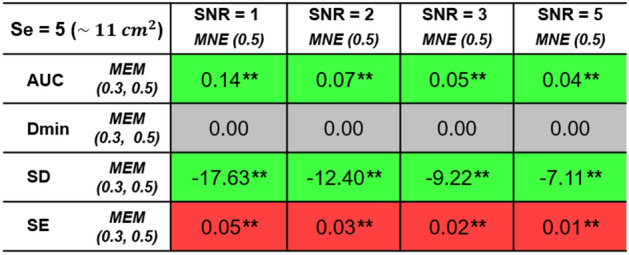
Reconstruction performance comparison of MEM and MNE with different SNR levels.

Median of paired difference of validation metric (i.e. AUC, Dmin, SD and SE) values of $Se = 5$ are presented in the table following the SNR increase from 1 to 5. ** indicates corrected $p<0.001$.

### Illustration of MEM and MNE reconstructions on real fNIRS data

**Figure 7:**
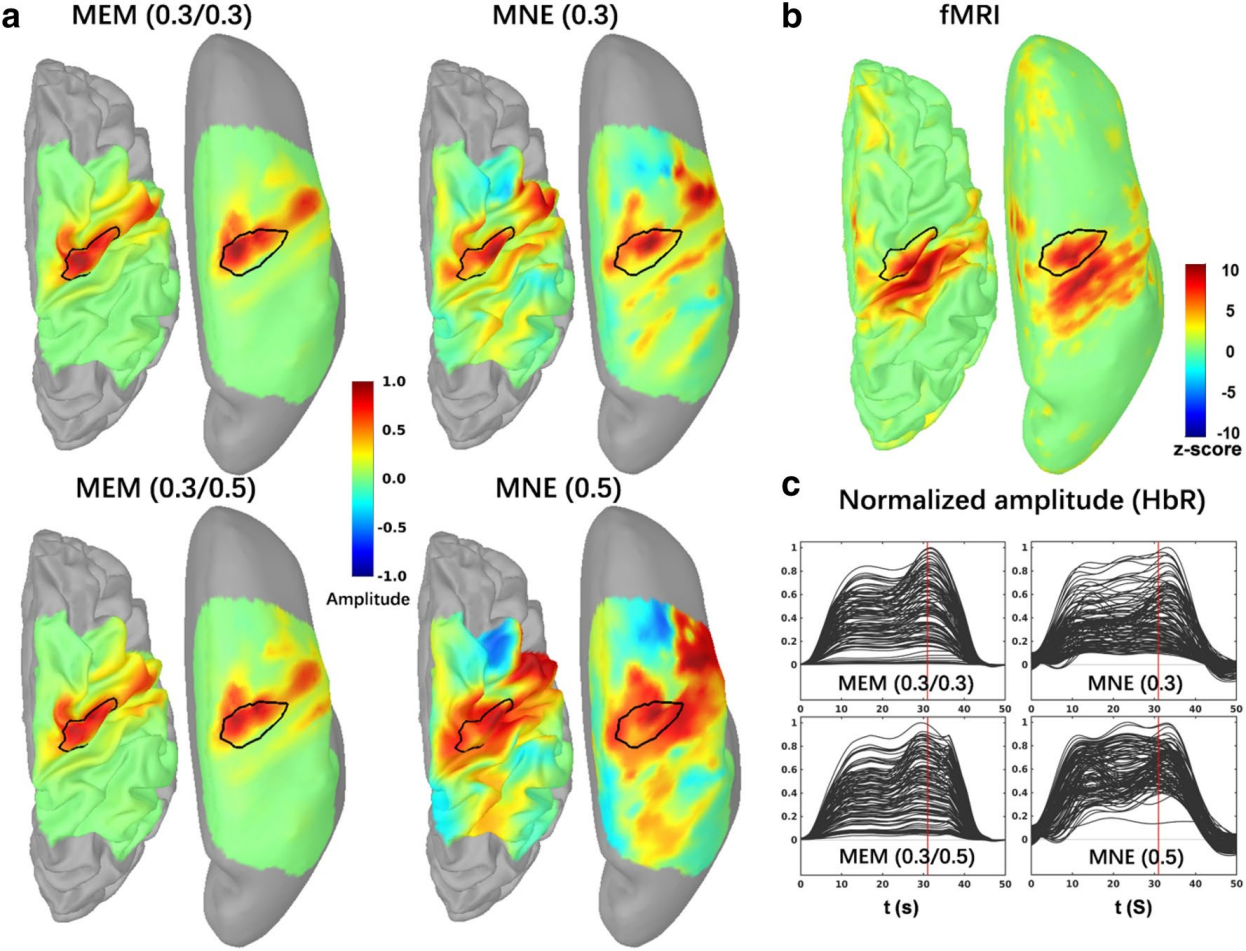
Application of MEM versus MNE reconstruction of HbR during a finger tapping task on one healthy subject. (**a**) Reconstructed maps of HbR (e.g. $20\%$ inflation on the left and $100\%$ inflation on the right side.) from MEM and MNE with different depth compensations. Each map was normalized by its own global maximum. (**b**) fMRI Z-map results projected along the cortical surface. (**c**) Reconstructed time courses of HbR within the hand knob region from MEM and MNE. Note that the hand knob region, represented by the black profile, was also matched well with the mean cluster of fMRI activation map on primary motor cortex. No statistical threshold was applied on fNIRS reconstructions. Figure created by Brainstorm^59^ using the NIRSTORM plugin developed by our team (https://github.com/Nirstorm).

For all finger tapping fNIRS data considered in our evaluations, two wavelength (i.e., 685*nm* and 830*nm*) were reconstructed first and then converted to HbO/HbR concentration changes along cortical surface using the standard absorption coefficients for each wavelength and each hemoglobin chromophore (HbO, HbR), as reported in our previous publications^19,47,70^. All the processes from fNIRS preprocessing to 3D reconstruction were completed in Brainstorm^59^ using the NIRSTORM plugin developed by our team (https://github.com/Nirstorm). For full double density montage (montage 1), reconstructed HbR amplitudes were reversed to positive phase and normalized to their own global maximum, to facilitate comparisons. In Fig. 7a, we showed the reconstructed HbR maps at the peak of the time course (i.e., 31*s*) for MEM and MNE by considering the 4 depth weighted versions, previously evaluated, i.e., MEM(0.3, 0.3), MEM(0.3, 0.5), MNE(0.3) and MNE(0.5). The two depth weighted versions of MEM clearly localized well the “hand knob” region, while exhibiting very little false positives in its surrounding. On the other hand, both depth weighted version of MNE clearly overestimated the size of the hand knob region and were also exhibiting some distant possibly spurious activity. The fMRI Z-map obtained during the corresponding fMRI task is presented on Fig. 7b, after projection of the volume Z-map on the cortical surface. Fig. 7c showed the time courses within the region of interest representing the “hand knob”. Each curve represents the reconstructed time course of one vertex of the hand knob region and the amplitude were normalized by the peak value within the whole region. Further illustrations of MEM and MNE performance on finger tapping for 5 subjects with montage 2 are presented in Supplementary material S4.

## Discussion

In the present study, we first adapted the MEM framework in the context of 3D fNIRS reconstruction and extensively validated its performance. The spatial performance of reconstructions can be considered in two aspects, 1) correctly localizing the peak of the reconstructed map close enough to the ground truth area, 2) accurately recovering the spatial extent of the generator. According to our comprehensive evaluations of the proposed depth-weighted implementations of MEM and MNE methods, accurate localization was overall not difficult to achieve as suggested by our results using Dmin metric. Almost all methods provided median value of Dmin to be 0 mm in all simulation conditions except for the lowest $SNR = 1$ condition where more localization error was found. On the other hand, recovering the actual spatial extent of the underlying generator is actually the most challenging task in fNIRS reconstruction. When considering the results of MNE on both realistic simulations and real finger tapping tasks, either from visual inspection (Figs. 4, 7 and S4) or quantitative evaluation by SD (Fig. 3, Table 1 and supplementary Sect. S2), we found that MNE overall reconstructed well the main generator but largely overestimated the size of the underlying generator. MEM was specifically developed, in the context of EEG/MEG source imaging, as a method able to recover the spatial extent of the underlying generators, which has been proved not to be the case for MNE-based approaches^33,36–38,40^. A recent review^71^ in the context of EEG/MEG source imaging also suggests that the Bayesian approach with sparsity constraints is required to accurately estimate the spatial extent. These important properties of MEM were successfully demonstrated in our results on fNIRS reconstructions. These excellent performances on spatial accuracy and sensitivity to the spatial extent of the underlying generators, as quantified using Dmin, AUC and SD metrics, were reliable for different sizes and depths of simulated generators, and for real finger tapping fNIRS data as well.

In this study, we performed a detailed evaluation of depth-weighted MNE reconstruction and we also proposed for the first time a depth weighting strategy within the MEM framework, by introducing two parameters: $\omega _1$ acting on scaling the source covariance matrix, and $\omega _2$ tuning the initialization of the reference for MEM. When compared to depth weighted MNE, the MEM framework demonstrated its ability to reconstruct, different depth of focal generators as well as larger size generators, exhibiting excellent spatial accuracy to recover generators of different depths and spatial extent, as quantified using large AUC values (e.g., high AUC values) and few false positives (e.g., low SD values, see Fig. 5). When considering deeper focal generators ($depth > 2 \, {\text {cm}}$), MEM(0.5, 0.5) clearly outperformed all other methods (see AUC and SD values in Fig. 5). In summary, for a large range of depths and spatial extents of the underlying generators, MEM methods exhibited accurate results (large AUC values) and less false positives (lower SD values) when compared to MNE methods. In practice, we would suggest to consider either $\omega _2=0.3$ or 0.5 for the initialization of MEM in all cases and only tune $\omega _1$. This is due to the fact that MNE(0.3 or 0.5) provided a generally good reconstruction with larger true positive rate in most scenarios, therefore providing MEM an accurate reference model, $d\nu (x)$, to start with. Even when considering the most focal simulated generators ($Se = 3$) case (see Figs. 3, 5 and Table 1), MEM(0.3, 0.3) and MEM(0.3, 0.5) were actually exhibiting very similar performances. Our suggestion to tune $\omega _1$ and $\omega _2$ parameters was actually further confirmed when considered results obtained from real data. For both montages, MEM(0.3, 0.3) results in excellent spatial agreement with fMRI Z-maps. Note that depth weighting was also considered in DOT studies using MNE^9,12,14,15,22,53^ and a hierarchical Bayesian DOT algorithm^11,29,30^. A spatially-variant regularization parameter $\beta$ was added to a diagonal regularization matrix featuring the sensitivity of every generator (forward model), and the value of $\beta$ was tuned according to the sensitivity value of a certain depth. In practice, this strategy would result in similar depth compensation as ours, but we preferred the depth weighting parameter $\omega$ which mapped the amount of compensation from 0 to 1 [as described in Eq. (3)] for easier interpretation and comparison. This is also a standard procedure introduced in EEG/MEG source localization studies^51,54^. Finally, using the depth weighted MNE solution as the prior is a common consideration in Hierarchical Bayesian framework based fNIRS reconstructions^11,29,30^.

Another important contribution of this study was that we improved the temporal accuracy time courses estimated within the MEM framework, resulting in similar temporal accuracy the one obtained with MNE. The largest significant SE difference between MEM and MNE was only 0.02 for $Se = 3$ and 0.01 for $Se = 5$. Corresponding time course estimations are also reported for MEM and MNE in real data (Figs. 7 and S4), suggesting again very similar performances. For instance, SE between MEM and MNE HbO time course was estimated as 0.02 for *Sub*05 in Fig. S4. Moreover, we found no significant SE differences between MEM and MNE for more extended generators (Se = 7,9). These findings are important considering that MNE is just a linear projection therefore the shape of the reconstruction will directly depend on the averaged signal at the channel level. On the other hand, MEM is a nonlinear technique, applied at every time sample, which is not optimized for the estimation of resulting time courses.

To further investigate the effects of the amount of realistic noise in our reconstructions on both reconstruction methods, we performed the comparisons along 4 different SNR levels, i.e., $SNR = 1,2,3,5$. As shown in Fig. 6 and Table 2, we found that MEM was overall more robust than MNE when dealing with simulated signals at lower SNR levels. This is actually a very important result since when reconstructing HbO/HbR responses, one has to consider at least two $\Delta$OD of two different wavelengths exhibiting different SNR levels. For the simulation results, we reported reconstruction results obtained from 830*nm* data, whereas when considering real data (Figs. 7 and S4), we had to convert the reconstruction absorption changes at 685*nm* and 830*nm* into HbO/HbR concentration changes. Therefore, our final results were influenced by the SNR of all involved wavelengths. fNIRS is inherently sensitive to inter-subject variability^72^, as also suggested in our application on real data presented in Fig. S4. Data from *Sub*05 were exhibiting a good SNR level and therefore both MEM and MNE reconstructed accurately the main cluster of the activation, while MNE presented more spatial spread and false positive activation outside the fMRI ROI. When considering subjects for whom we obtained lower SNR data, e.g., *Sub*02 and *Sub*03, MEM still recovered an activation map similar to fMRI map. In those cases, MNE not only reported suspicious activation pattern but also incorrectly reconstruct the peak amplitude outside the fMRI ROI. Our results suggesting MEM robustness in low SNR conditions for DOT are actually aligned with similar findings suggested for EEG/MEG source imaging, when considering source localization of single trial data^39,42^.

To perform a detailed evaluation of our proposed fNIRS reconstructions methods, we developed a fully controlled simulation environment, similar to the one proposed by our team to validate EEG/MEG source localization methods^33,36,38^. The fNIRS resting state data, acquired by the same montage (montage1) and underwent the same preprocessing as conducted for the real data, was added to the simulated true hemodyanmic response for each channel. Indeed such environment provided us access to a ground truth, which is not possible when considering real fNIRS data set. Previous studies validated tomography results^11,22^ by comparing with fMRI activation map which can indeed be considered as a ground truth, but only for well controlled and reliable paradigms. Since fMRI also measures a signal of hemodynamic origin, it is reasonable to check the concordance between fMRI results and DOT reconstructions. Therefore, as preliminary illustrations, we also compared our MEM and MNE results to fMRI Z-maps obtained during finger tapping tasks on 6 healthy participants (Figs. 7 and S4), suggesting overall excellent performances on spatial accuracy (by qualitative visual inspections) of MEM when compared to MNE. Further quantitative comparison between fMRI and fNIRS 3D reconstruction, on well controlled finger tapping data using MEM and MNE, was conducted in our follow up study^73^.

Several software packages have been proposed to provide fNIRS reconstruction pipelines, as for instance NeuroDOT^22,74^, AtlasViewer^75^ and fNIRS-SPM^76^. To ensure an easy access of our MEM methodology to the fNIRS community, we developed and released a fNIRS processing toolbox - NIRSTORM (https://github.com/Nirstorm), as a plugin of Brainstorm software^59^, which is a renown software package dedicated for EEG/MEG analysis and source imaging. Our package NIRSTORM offers standard preprocessing, analysis and visualization as well as more advanced features such as personalized optimal montage design, access to forward model estimation using MCXlab^64,65^ and the MNE and MEM implementations considered in this study.

Previously, Tremblay et al.^23^ had comprehensively compared a variety of fNIRS reconstruction methods using large number of realistic simulations. Since introducing MEM was our main goal of this study, we did not consider such wide range of methodological comparisons. We decided to carefully compare MEM with MNE since MNE remains the main method considered for DOT, and is available in several software packages. As suggested in Tremblay et al.^23^, DOT reconstruction methods based on Tikhonov regularization, such as least square regularization in MNE, usually allow great sensitivity, but performed poorly in term of spatial extent - largely overestimating the size of the underlying generator. On the other hand, L1-based regularization^24–27^ could achieve more focal solutions with high specificity but much lower sensitivity. As demonstrated in our results, the proposed MEM framework allows reaching good sensitivity and accurate reconstruction of the spatial extent of the underlying generator. Bayesian model averaging (BMA) originally proposed for EEG source imaging by Trujillo-Barreto et al.^77^, also allows accurate DOT reconstructions with less false positives when compared to MNE. Similarly, we carefully compared MEM to Bayesian multiple priors approaches in Chowdhury et al.^33^ in the context of MEG source imaging. Comparing MEM with more advanced DOT reconstruction methods, including also the one proposed by Yamashita et al.^11^, would be of great interest but was out of the scope of this study.

Considering the main contribution of this study was to introduce and adapt the MEM framework for 3D fNIRS reconstruction, we decided to first carefully evaluate the performance of MEM using well controlled realistic simulations. We also included few real data set reconstructions to illustrate the performance of the MEM reconstruction, whereas quantitative evaluation of MEM reconstructions on a larger database were conducted in our follow-up study^73^. In this complementary study, we conducted both individual and group-level quantitative comparisons between fNIRS reconstructed maps and fMRI activation maps, both at the individual and group levels. In agreement with our detailed evaluation on realistic simulations, our results reported in^73^ also showed that MEM provided better spatial accuracy than MNE, while both methods offered similar temporal accuracy when reconstructing HbO/HbR concentration changes evoked by finger-tapping. In previous reported studies^12–14,22,69^, a high density montage was considered which was proved to be able to provide high spatial resolution and robustness to low SNR conditions^13^, evaluating the performance of MEM when considering high density fNIRS montage would be of great interest but was out of the scope of this present study.

## Conclusion

In this study, we introduced a new fNIRS reconstruction method entitled Maximum Entropy on the Mean (MEM). We first implemented depth weighting into MEM framework and improved its temporal accuracy. To carefully validate the method, we applied a large number ($n=4000$) of realistic simulations with various spatial extents and depths. We also evaluated the robustness of the method when dealing with low SNR signals. The comparison of the proposed method with the widely used depth weighted MNE was performed by applying four different quantification validation metrics. We found that MEM framework provided accurate and robust reconstruction results, relatively stable for a large range of spatial extents, depths and SNRs of the underlying generator. Moreover, we implemented the proposed method into a new fNIRS processing plugin - NIRSTORM in Brainstorm software to provide the access of the method to users for applications, validations and comparisons.

## Supplementary Information

Supplementary Information 1.

Supplementary Information 2.
