# Supplementary material for: Diffuse optical reconstructions of functional near infrared spectroscopy data using maximum entropy on the mean
Source: Sci Rep. 2022 Feb 10;12:2316. doi: 10.1038/s41598-022-06082-1 (PMC8831678; doi:10.1038/s41598-022-06082-1)
Supplement: Supplementary file 2 — Supplementary Information 2. [file 41598_2022_6082_MOESM2_ESM.pdf]

Supplementary material

S1. Validation metrics

Here is a detailed description of the four validation metrics considered in our evaluation. Except the shape error (SE), other metrics were all calculated at the time instant τ when the simulated ΔOD time course reached its peak value (e.g. $12.2s$ after onset).

Area Under the Receiver Operating Characteristic (ROC) curve (AUC) was used to assess overall detection accuracy of the reconstruction methods. We used a specific version of AUC that has been proposed in[1] in order not to bias results towards false positives. In further details, ROC curves were generated by plotting the sensibility of the detection as a function of 1-specificity, while thresholding the normalized reconstruction map from 0 to 1 with a certain step value. In the context of source reconstruction, especially when the generator is focal, the region of true positive is usually much smaller than the region of true negative, whereas non-biased AUC evaluation would require to sample the same amount of active and inactive generators. To overcome this possible bias, we considered a ROC evaluation using the same number of active and inactive generators that were randomly sampled within two different regions: 1) AUC_{close}: inactive generators were sampled within the immediate spatial neighborhood of the ground truth; and 2) AUC_{far}: inactive generators were sampled within the local maxima of the reconstructed activity located far from the ground truth. The final AUC was then the average of AUC_{close} and AUC_{far}.

Minimum geodesic distance (Dmin) was represented by the geodesic distance, following the circumvolutions of the cortical surface, of the vertex that exhibited maximum of reconstructed activity to the border of the 'generator'. It should be 0 when the peak of the reconstruction map was located inside the simulated cortical region.

Spatial Dispersion (SD) assessed the spatial spread of the estimated 'generator' distribution and the localization error using Eq.S1. The ideal value (i.e. $SD = 0mm$), was achieved when no activation was reconstructed outside the theoretical 'generator'. The larger the SD was, the more spatially spread were the reconstructed maps.

$$SD = \sqrt{\frac{\sum_{i=1}^{K}\left(min_{j\in\Theta}(D^2(i,j))\hat{X}^2(i,\tau)\right)}{\sum_{i=1}^{K}\left(\hat{X}^2(i,\tau)\right)}} \tag{S1}$$

where $min_{j\in\Theta}(D^2(i,j))$ is the minimum Euclidean distance between the vertex i to the vertex j which is located inside the simulated 'generator' (Θ). $\hat{X}^2(i,\tau)$ is the power of the amplitude of reconstructed time course on vertex i at time τ. K is the total number of vertices within the reconstruction field of view.

Shape error(SE) evaluated the temporal accuracy of the reconstruction. Reconstructed time courses within the simulation were averaged and normalized. The root mean square of the difference between this time course and the normalized theoretical time course was estimated and denoted as SE in Eq.S2 as introduced in[2]

$$SE = \sqrt{\frac{1}{T}\sum_{t}^{T}\left(\frac{X_{th}(t)}{max(|X_{th}(t)|)} - \frac{mean_{j\in\Theta}(\hat{X}(j,t))}{max(|mean_{j\in\Theta}(\hat{X}(j,t))|)}\right)^2} \tag{S2}$$

where T is length of the time course. $X_{th}(t)$ is the theoretical time course of the simulation. $mean_{j\in\Theta}(\hat{X}(j,t))$ is the averaged mean of the reconstructed time courses within the 'generator'.

S2. Effects of depth weighting on MNE

We first investigated the effects of depth weighting factor ω_2 selection for depth weighted MNE. To do so, we evaluated spatial and temporal performances of DOT reconstruction. As presented in Fig.S1, we compared depth weighted MNE using depth weighting factors $\omega_2 = 0, 0.1, 0.3, 0.5, 0.7, 0.9$ in superficial seeds case. In general, $\omega_2 = 0.3$ and 0.5 provided overall the most accurate results (i.e. median $AUC > 0.8$ and $Dmin = 0mm$). For focal generators(i.e. $Se = 3, 5$), $\omega_2 = 0.3$ performed better than $\omega_2 = 0.5$ considering it was providing significantly lower SD. However, in extended generators (i.e. $Se = 7, 9$), reconstructions with $\omega_2 = 0.5$ were exhibiting more accurate results, consisting in significantly positive AUC difference (0.05 and 0.08, $p < 0.001$) and significantly positive SD difference (2.24 and 2.06, $p < 0.001$). $\omega_2 = 0$ and 0.1 only provided AUC higher than 0.8 in the case of $Se = 3$, whereas $\omega_2 = 0.7$ and 0.9 failed in all cases and even the median values of Dmin were significantly larger (median values around 2-3 cm) than other cases. Based on these results, we decided to consider only the depth weighting values $\omega_2 = 0.3$ and 0.5 for depth weighting MNE in the comparisons with with MEM reconstructions.

S3. MEM v.s. MNE with realistic simulations involving middle and deep seeds

In Fig.S2 and Table.S1, we are presenting the comparison of MEM and MNE in middle seeds case. First of all, we found that more depth compensation was required to provide good reconstructions in all scenarios. Thus, MEM(0.5, 0.5) was compared to

the best of MNE - MNE(0.5). Non-significant AUC and Dmin differences were found between them. However, MEM(0.5,0.5) provided significant lower SD than MNE(0.5), median value of difference of $SD = -5.33, -4.80, -5.00, -4.95$, $p < 0.001$ for $Se = 3,5,7,9$ respectively. Fig.S3 and Table.S2 are presenting the comparison of MEM and MNE in the comparison of them in deep seeds case. Similarly, no significant AUC and Dmin differences were found. MEM(0.5,0.5) provided significant lower SD than MNE(0.5), median value of difference of $SD = -6.39, -6.33, -6.97, -5.52$, $P < 0.001$ for $Se = 3,5,7,9$ respectively. For temporal performance in these two cases, similar to Fig.3 in the manuscript, MNE(0.5) gave significant lower SE (-0.01 or -0.02, $p < 0.001$) than MEM when $Se = 3,5$ (small difference). No significant different SE was found in $Se = 7,9$.

S4. Validation of fNIRS reconstruction using optimal montage

A personalized optimal montage (**Montage 2**, see Fig.S4) following the methodology we previously reported in Machado et al.[3] was applied to validate the fNIRS reconstruction results. First, the hand knob within right primary motor cortex was drawn manually along the cortical surface and defined as a target region of interest (ROI) using the Brainstorm software[4]. Then we applied optimal montage estimation[3,5] in order to estimate personalized montages, built to maximize a priori fNIRS sensitivity and spatial overlap between channels with respect to the target ROI. To ensure good spatial overlap between channels for local 3D reconstruction, we constructed personalized optimal montages composed of 3 sources and 15 detectors (see Fig.S4b). The source-detector distance was set to vary from $2cm$ to $4.5cm$ and each source was constrained such that it has to create channels with at least 13 detectors. Finally, we also manually added 1 proximity channel, located at the center of the 3 sources. Five subjects underwent fNIRS acquisitions with personalized optimal montage during a similar finger tapping task as the one for montage 1, in which 20 blocks were acquired by alternating a task (period of $10s$) and a resting state period ranging from $30s$ to $60s$.

fNIRS reconstruction results obtained on 5 subjects for acquisition involving personalized optimal fNIRS montage (montage 2) and corresponding fNIRS reconstructions are presented in Fig.S4. For every subject, fMRI Z-maps are presented along the left hemisphere only and thresholded at $Z > 3.1$ ($p < 0.01$, corrected using Gaussian random field theory), The most significant fMRI cluster along M1 and S1 was delineated using a black profile. Reconstruction maps at the corresponding HbO/HbR peaks are then presented. Similar accuracy between MEM and MNE, with good overlap with fMRI results, were found for subjects 4 and 5, while MNE was overestimating the spatial extent of the generator. For subject 1, 2 and 3, MNE exhibited poor spatial correspondence with fMRI results. Averaged reconstructed time courses within the fMRI main cluster region are shown with standard deviation as the error bar. Comparing to simulations results, MEM exhibited overall very similar time course estimations than MNE in all cases. Considering the task duration was $10s$, the reconstructed peak timing of HbO/HbR appeared accurately within the range of $10s$ to $20s$.

References

1. Grova, C. *et al.* Evaluation of EEG localization methods using realistic simulations of interictal spikes. *NeuroImage* **29**, 734–753, DOI: 10.1016/j.neuroimage.2005.08.053 (2006).

2. Chowdhury, R. A., Lina, J. M., Kobayashi, E. & Grova, C. MEG Source Localization of Spatially Extended Generators of Epileptic Activity: Comparing Entropic and Hierarchical Bayesian Approaches. *PLoS ONE* **8**, DOI: 10.1371/journal.pone.0055969 (2013).

3. Machado, A. *et al.* Optimal positioning of optodes on the scalp for personalized functional near-infrared spectroscopy investigations. *J. Neurosci. Methods* **309**, 91–108, DOI: 10.1016/J.JNEUMETH.2018.08.006 (2018).

4. Tadel, F., Baillet, S., Mosher, J. C., Pantazis, D. & Leahy, R. M. Brainstorm: A user-friendly application for MEG/EEG analysis. *Comput. Intell. Neurosci.* **2011**, 879716, DOI: 10.1155/2011/879716 (2011).

5. Machado, A., Marcotte, O., Lina, J. M., Kobayashi, E. & Grova, C. Optimal optode montage on electroencephalography/functional near-infrared spectroscopy caps dedicated to study epileptic discharges. *J. Biomed. Opt.* **19**, 026010, DOI: 10.1117/1.JBO.19.2.026010 (2014).

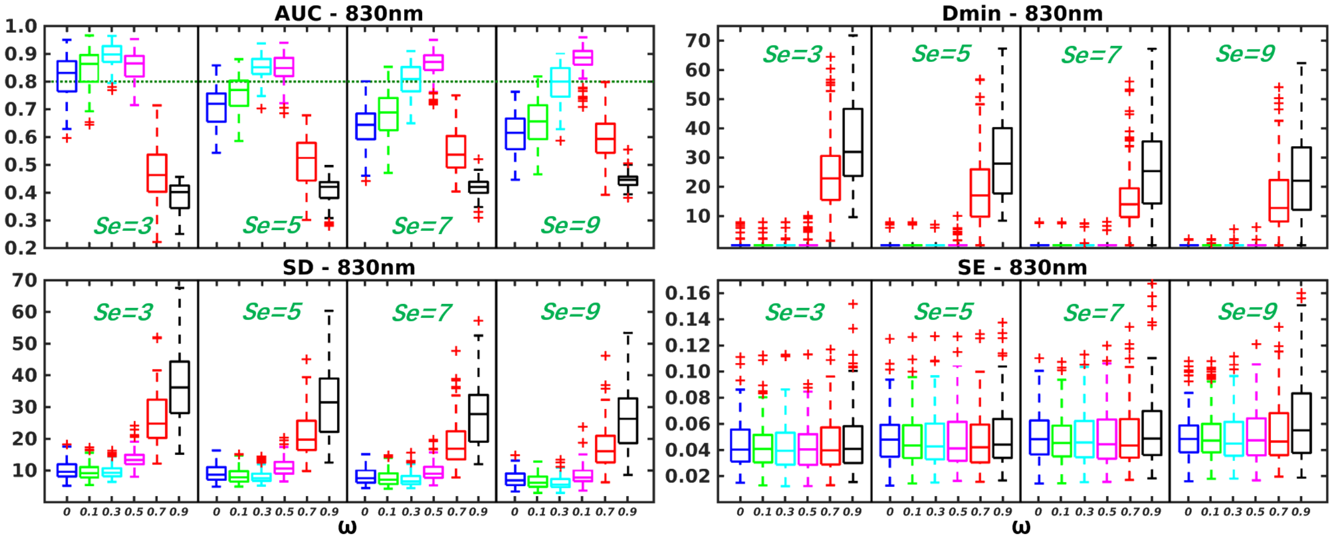

Fig.S1 Evaluation of the performances of depth weighted MNE for different depth weighting factors $\omega = 0, 0.1, 0.3, 0.5, 0.7$ and 0.9. Distribution of validation metrics (AUC, Dmin , SD and SE) are displayed using boxplot representations, for simulations involving superficial seeds only and for spatial extents $Se = 3, 5, 7$ and 9.

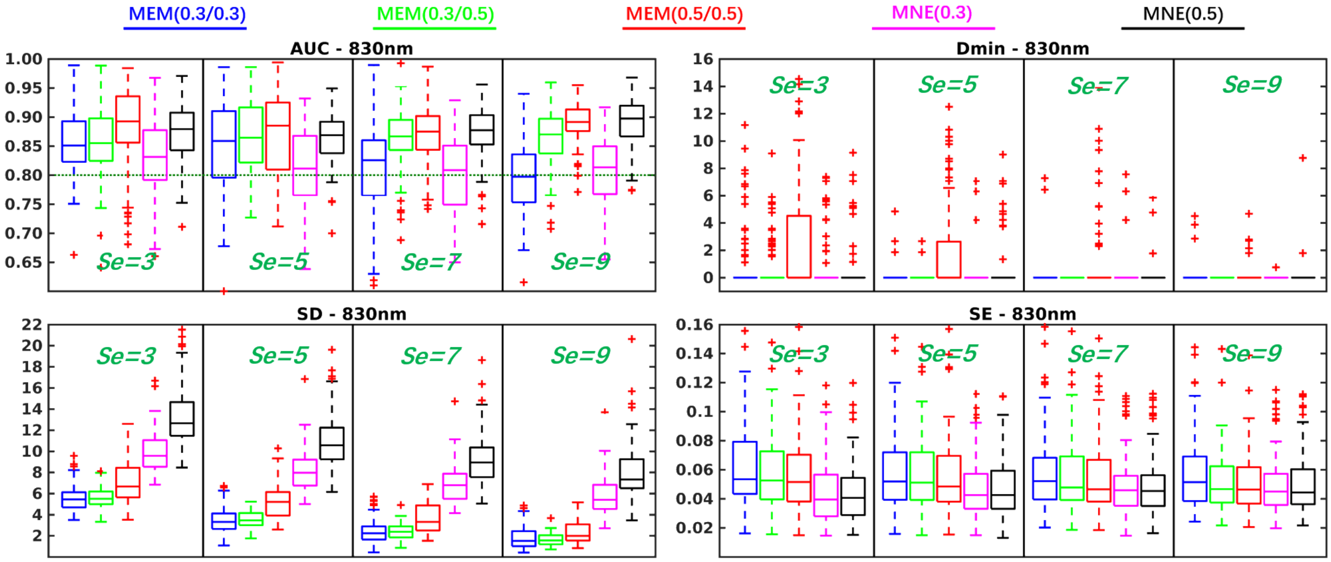

Fig.S2 Evaluation of the performances of MEM and MNE using realistic simulations involving middle seeds for different spatial extent ($Se = 3, 5, 7, 9$). Boxplot representation of the distribution of four validation metrics for three depth weighted strategies of MEM and two depth weighted strategies of MNE, namely: MEM(0.3, 0.3) in blue, MEM(0.3, 0.5) in green, MEM(0.5, 0.5) in red, MNE(0.3) in magenta and MNE(0.5) in black. Results were obtained after DOT reconstruction of $830nm\ \Delta OD$.

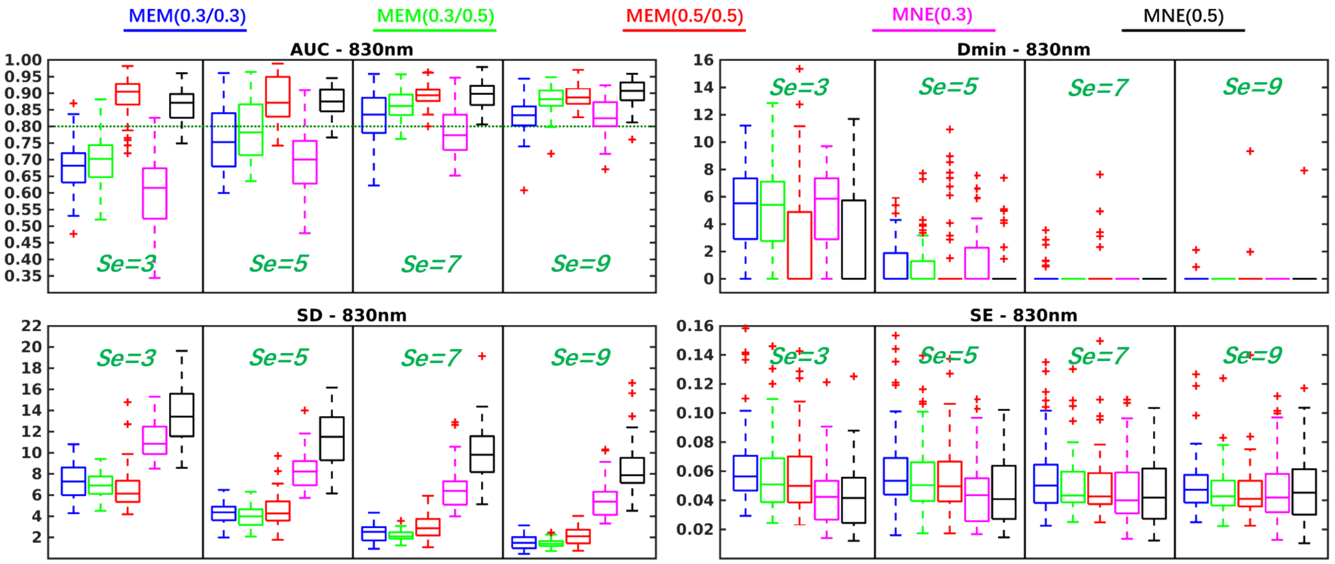

Fig.S3 Evaluation of the performances of MEM and MNE using realistic simulations involving deep seeds for different spatial extent ($Se = 3, 5, 7, 9$). Boxplot representation of the distribution of four validation metrics for three depth weighted strategies of MEM and two depth weighted strategies of MNE, namely: MEM(0.3, 0.3) in blue, MEM(0.3, 0.5) in green, MEM(0.5, 0.5) in red, MNE(0.3) in magenta and MNE(0.5) in black. Results were obtained after DOT reconstruction of 830nm ΔOD.

Middle Seeds		Se = 3		Se = 5		Se = 7		Se = 9	
		MNE (0.3)	*MNE (0.5)*	*MNE (0.3)*	*MNE (0.5)*	*MNE (0.3)*	*MNE (0.5)*	*MNE (0.3)*	*MNE (0.5)*
AUC	*MEM (0.3, 0.3)*	0.03**	-0.03	0.03**	0.00	0.02	-0.05**	-0.02	-0.10**
	MEM (0.3, 0.5)	0.03**	-0.03	0.05**	0.01	0.05**	-0.01	0.05**	-0.02**
	MEM (0.5, 0.5)	0.06**	0.02	0.07**	0.01	0.07**	-0.01	0.08**	0.00
Dmin	*MEM (0.3, 0.3)*	0.00	0.00	0.00	0.00	0.00	0.00	0.00	0.00
	MEM (0.3, 0.5)	0.00	0.00	0.00	0.00	0.00	0.00	0.00	0.00
	MEM (0.5, 0.5)	0.00	0.00	0.00	0.00	0.00	0.00	0.00	0.00
SD	*MEM (0.3, 0.3)*	-4.05**	-7.21**	-4.27**	-7.25**	-4.10**	-6.40**	-3.58**	-5.43**
	MEM (0.3, 0.5)	-4.00**	-7.06**	-4.09**	-6.90**	-3.96**	-6.40**	-3.65**	-5.45**
	MEM (0.5, 0.5)	-2.54**	-5.33**	-2.46**	-4.80**	-2.85**	-5.00**	-3.08**	-4.95**
SE	*MEM (0.3, 0.3)*	0.02**	0.02**	0.01**	0.01**	0.01**	0.01*	0.00	0.01
	MEM (0.3, 0.5)	0.01**	0.01**	0.01**	0.01**	0.00	0.00	0.00	0.00
	MEM (0.5, 0.5)	0.01**	0.01**	0.01**	0.01*	0.00	0.00	0.00	0.00

Table.S1 Wilcoxon signed rank test results of reconstruction performance comparison of MEM and MNE in middle seeds case. Median values of paired difference are presented in the table. p values were corrected for multiple comparisons using Bonferroni correction, * indicates $p < 0.01$ and ** represents $p < 0.001$. Median of the paired difference of each validation metrics is color coded as follows: green: MEM is significantly better than MNE, red: MNE is significantly better than MEM and gray: non-significance.

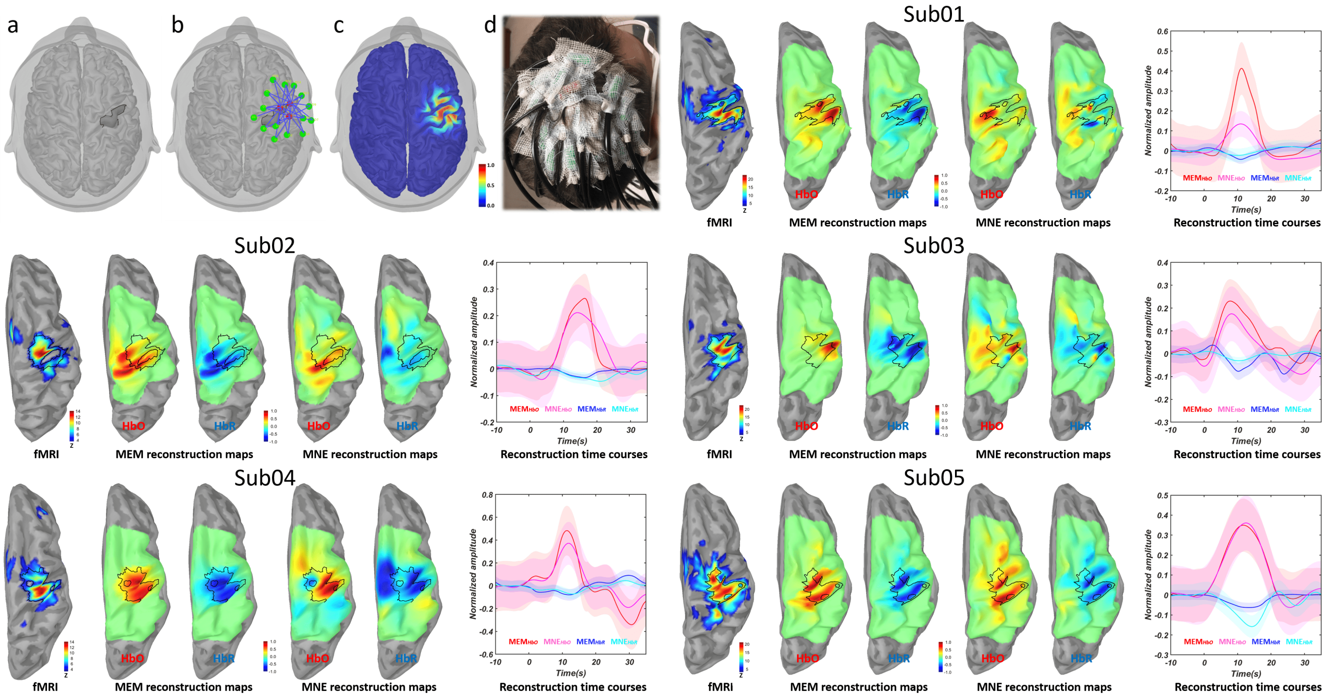

Fig.S4 Personalized fNIRS montage and comparisons between MEM and MNE reconstructions with respect to fMRI Z-map at individual level. a) the region of interest defined as the "hand knob", b) optimal montage targeting the ROI consisting 3 sources (red) and 15 detectors(green) and one proximity (in the center of sources not shown), c) normalized sensitivity profile of the optimal montage which calculated as the sum of all channel's sensitivity along the cortical surface, d) optimal montage glued on the scalp of the one subject, using collodion. fMRI Z-map of each subject during finger tapping task (threshold with $Z > 3.1$, Bonferroni corrected), black profile represents the main cluster along M1 and S1. MEM reconstruction maps at the corresponding HbO/HbR peak times, using depth weighted option $0.3, 0.3$. MNE reconstruction maps, at the corresponding HbO/HbR peak times, using depth weighted option 0.3. Reconstructed time courses within the black profile, solid lines represent the main time courses and the shade areas represent standard deviation within the region of interest. Reconstructed time courses were normalized by the maximum amplitude, for each method respectively, before averaging.

Deep Seeds		Se = 3		Se = 5		Se = 7		Se = 9	
		MNE (0.3)	*MNE (0.5)*	*MNE (0.3)*	*MNE (0.5)*	*MNE (0.3)*	*MNE (0.5)*	*MNE (0.3)*	*MNE (0.5)*
AUC	***MEM (0.3, 0.3)***	0.08**	-0.20**	0.06**	-0.13**	0.05**	-0.08**	0.00	-0.07**
	MEM (0.3, 0.5)	0.09**	-0.17**	0.08**	-0.08**	0.08**	-0.03*	0.05**	-0.02
	MEM (0.5, 0.5)	0.29**	0.03	0.18**	-0.01	0.13**	-0.01	0.06**	-0.01
Dmin	***MEM (0.3, 0.3)***	0.00	2.91	0.00	0.00	0.00	0.00	0.00	0.00
	MEM (0.3, 0.5)	0.00	2.16	0.00	0.00	0.00	0.00	0.00	0.00
	MEM (0.5, 0.5)	-3.53	0.00	0.00	0.00	0.00	0.00	0.00	0.00
SD	***MEM (0.3, 0.3)***	-3.73**	-6.25**	-3.65**	-7.37**	-3.51**	-7.39**	-3.46**	-6.21**
	MEM (0.3, 0.5)	-4.00**	-6.61**	-3.83**	-7.54**	-3.95**	-7.63**	-3.82**	-6.50**
	MEM (0.5, 0.5)	-4.56**	-6.39**	-3.73**	-6.33**	-3.10**	-6.97**	-3.33**	-5.52**
SE	***MEM (0.3, 0.3)***	0.02**	0.02**	0.02**	0.02**	0.01*	0.01	0.01	0.00
	MEM (0.3, 0.5)	0.01**	0.01**	0.01*	0.01**	0.01	0.01	0.00	0.00
	MEM (0.5, 0.5)	0.01*	0.01**	0.01*	0.01*	0.00	0.01	0.00	0.00

Table.S2 Wilcoxon signed rank test results of reconstruction performance comparison of MEM and MNE in deep seeds case. Median values of paired difference are presented in the table. p values were corrected for multiple comparisons using Bonferroni correction, * indicates $p < 0.01$ and ** represents $p < 0.001$. Median of the paired difference of each validation metrics is color coded as follows: green: MEM is significantly better than MNE, red: MNE is significantly better than MEM and gray: non-significance.
